# A single generation in the wild increases fitness for descendants of hatchery‐origin Chinook salmon (*Oncorhynchus tshawytscha*)

**DOI:** 10.1111/eva.13678

**Published:** 2024-04-11

**Authors:** David I. Dayan, Nicholas M. Sard, Marc A. Johnson, Cristín K. Fitzpatrick, Ryan Couture, Kathleen G. O'Malley

**Affiliations:** ^1^ State Fisheries Genomics Lab, Coastal Oregon Marine Experiment Station, Department of Fisheries, Wildlife, and Conservation Sciences, Hatfield Marine Science Center Oregon State University Newport Oregon USA; ^2^ Department of Biological Sciences State University of New York‐Oswego Oswego New York USA; ^3^ Native Fish Conservation and Recovery, Oregon Department of Fish and Wildlife Salem Oregon USA; ^4^ Oregon Department of Fish and Wildlife Corvallis Oregon USA

**Keywords:** captive‐rearing, reintroduction, relative reproductive success, translocation, trap‐and‐haul

## Abstract

Reintroduction is an important tool for the recovery of imperiled species. For threatened Pacific salmonids (*Oncorhynchus* spp.) species, hatchery‐origin (HOR) individuals from a nearby source are often used to reestablish populations in vacant, historically occupied habitat. However, this approach is challenged by the relatively low reproductive success that HOR Pacific salmonids experience when they spawn in the wild, relative to their natural‐origin (NOR) counterparts. In this study, we used genetic parentage analysis to compare the reproductive success of three groups of adult Chinook salmon (*Oncorhynchus tshawytscha*) reintroduced above Cougar Dam on the South Fork McKenzie River, Oregon: HOR Chinook salmon from an integrated stock; first‐generation, wild‐born descendants (hereafter *F*_1_s) of Chinook salmon produced at the same hatchery; and NOR Chinook salmon that are presumed to have been produced below the dam, on the mainstem McKenzie River, or elsewhere and volitionally entered a trap below Cougar Dam. We found that *F*_1_s produced nearly as many adult offspring as NORs, and 1.8‐fold more adult offspring than HORs. This result suggests that, for the South Fork McKenzie reintroduction program, a single generation in the wild increases fitness for the descendants of HOR Chinook salmon. Although these results are encouraging, care must be taken before extrapolating our results to other systems.

## 1 INTRODUCTION

Reintroducing organisms into habitats from which they have been extirpated is a common strategy used to aid in the recovery of threatened and endangered taxa (Seddon et al., 2007, 2014). Reintroduction programs often seek to establish productive, self‐sustaining natural populations (IUCN/SSC, 2013). However, they frequently fail to achieve their recovery and conservation objectives (Bellis et al., 2019; Bubac et al., 2019; Fischer & Lindenmayer, 2000; Godefroid et al., 2011). Many factors contribute to reintroduction success, or failure, including suitability of the source population (Bubac et al., 2019; Cochran‐Biederman et al., 2015). Source populations must express appropriate phenotypes in the new habitat, and possess sufficient phenotypic and genetic diversity to avoid short‐term extinction risk and promote the adaptive potential of the reintroduced population (Furlan et al., 2020; Malone et al., 2018; Miller et al., 2009; Weeks et al., 2011).

Pacific salmon (*Oncorhynchus* spp.) have been extirpated from much of their historical range (Gustafson et al., 2007), including more than half of the historical habitat in the Upper Willamette and Lower Columbia River basins (Sheer & Steel, 2011). Reintroducing populations into these habitats has the potential to provide tremendous benefits to Pacific salmon, especially where access to functional habitat can be restored through active transport of individuals or removal of passage barriers. Such reintroductions reduce extinction risk directly by providing additional abundance and productivity, and indirectly by promoting habitat, life history, and genetic diversity (Anderson et al., 2014; Kock et al., 2020; Lusardi & Moyle, 2017; McClure et al., 2018). However, selecting the appropriate source population for Pacific salmon reintroductions is challenging. Managers must choose between transplanting individuals from extant natural populations, promoting natural recolonization through passage barrier removal, or releasing hatchery‐origin (HOR) individuals (Anderson et al., 2014; McClure et al., 2018). Extensive transplanting from extant natural populations often poses unacceptable risks to threatened or endangered populations (Anderson et al., 2014; McClure et al., 2018). Removal of passage barriers is often not feasible, precluding natural recolonization by volitional dispersal. Consequently, Pacific salmon reintroductions are frequently seeded with HOR individuals from nearby stocks (Anderson et al., 2014). These HOR individuals produce offspring in the wild that subsequently return to the target habitat as adults to spawn. However, potentially low reproductive success of HOR salmonids, as compared to that of natural‐origin (NOR) salmonids (i.e., relative reproductive success; RRS) (Araki, Ardren, et al., 2007; Araki, Cooper, & Blouin, 2007; Christie et al., 2014; Ford, 2002; Koch & Narum, 2021; Kostow et al., 2003; Theriault et al., 2011), can thwart efforts to establish self‐sustaining populations. Similarly, low RRS challenges supplementation programs that seek to provide demographic benefits to extant natural populations by spawning NOR salmonids in the hatchery and allowing their offspring to reproduce in the wild as adults (Berntson et al., 2011; Christie et al., 2012; Hess et al., 2012).

Following more than two decades of research, there are many point estimates of RRS across salmonid species, hatchery programs, and river basins (reviewed in Christie et al., 2014; Koch & Narum, 2021). These studies have revealed important patterns and suggested areas where more research is needed. Across species, reproductive success of HOR salmonids tends to be less than that of NOR individuals (i.e., RRS <1), but RRS also depends on habitat and hatchery broodstock characteristics, and the age, size, and sex of individual spawners (Christie et al., 2014; Koch & Narum, 2021). In Chinook salmon (*O. tshawytscha*), there is evidence that integrating 100% wild‐born spawners into the broodstock may ameliorate the fitness impacts of captive rearing (Hess et al., 2012; Janowitz‐Koch et al., 2019), although this result is not observed consistently across populations (Koch et al., 2022). In contrast, RRS of Chinook salmon is consistently less than one when broodstocks are segregated, or only partially integrated (Anderson et al., 2013; Evans et al., 2016; O'Malley et al., 2023; Sard et al., 2015; Sard, Johnson, et al., 2016; Williamson et al., 2010). To date, the literature has primarily focused on documenting potential fitness differences between HOR and NOR salmonids spawning in the wild, the pace at which these fitness differences, if present, accrue, and the role of contributing factors. Yet, overcoming the challenge posed to reintroduction and supplementation by low RRS of HOR individuals depends on whether there is a transgenerational increase in fitness among their wild‐born offspring.

To our knowledge, this question has only been explicitly addressed in three populations of salmonids. Araki et al. (2009) found that early generation, wild‐born descendants of HOR steelhead (*O. mykiss*) in the Hood River, Oregon, had lower reproductive success in the wild than wild‐born steelhead with no recent hatchery ancestry, but a re‐analysis of these data to account for variance and bias in RRS estimates could not reject the null hypothesis that RRS was equal to one (Kitada et al., 2011). In Chinook salmon from the McKenzie River, Oregon, we previously observed that first‐generation, wild‐born hatchery descendants produced more age‐0 offspring than HOR Chinook salmon from a partially integrated stock, and similar numbers to other wild‐born Chinook salmon in the same habitat across 2 years. But, we did not account for bias from fitness‐associated covariates, or estimate fitness using adult offspring (Banks et al., 2016). Finally, Nuetzel et al. (2023) found that early generation descendants from a partially integrated stock of Chinook salmon reintroduced to Lookingglass Creek, Oregon, produced more juvenile offspring, adult offspring, and juvenile grand‐offspring than HOR Chinook salmon spawning in the same habitat. This finding was consistent across multiple years and after accounting for fitness‐associated covariates (e.g., return day, body length, and sex). However, only HOR Chinook salmon and their descendants were reintroduced in this system, and reproductive success relative to wild‐born Chinook salmon from a natural population could not be evaluated.

In this study, we examine a Chinook salmon pedigree from a reintroduction program above Cougar Dam on the South Fork McKenzie River, Oregon, to evaluate how the source of reintroduced individuals influences reproductive success. Specifically, we test whether lower reproductive success of HOR Chinook salmon in the wild persists among wild‐born descendants of HOR Chinook salmon. We also compare the reproductive success of these wild‐born hatchery descendants born above the dam to that of NOR Chinook salmon born below the dam, the mainstem McKenzie River, or elsewhere that volitionally enter the trap at the base of Cougar Dam. While this latter group may or may not be reflective of a hypothetical locally adapted population from above Cougar Dam, it is representative of natural colonizers that might act to reestablish the population in absence of the dam, and provides important context for our results. Finally, we examine if the adult offspring produced by Chinook salmon reintroduced above Cougar Dam differed in two ecologically relevant phenotypes, age and size at maturity. We do not examine if differences in reproductive success, or the characteristics of adult offspring, are due to environmental or evolved genetic changes. We conclude by highlighting that care must be taken before extrapolating our results to other systems.

## 2 METHODS

### 2.1 Reproducible research

Detailed logs and all data from this study are available at a github repository, https://github.com/david‐dayan/mckenzie_naturalization, and archived at zenodo with a stable identifier (DOI: 10.5281/zenodo.8097487). An R notebook containing narrative logs of all analyses with integrated code, results, and commentary is available as a supplementary file (Appendix S2).

### 2.2 Study system

Spring Chinook salmon in the Upper Willamette River are listed as a threatened evolutionarily significant unit (ESU) under the US Endangered Species Act (ESA) (NMFS, 1999). The McKenzie River, a tributary of the Upper Willamette River, historically supported one of the largest populations of spring Chinook salmon and currently supports a large proportion of the naturally produced spring Chinook salmon in the upper Willamette Basin (Johnson & Friesen, 2010; McElhany et al., 2007). Spring Chinook salmon in the upper reaches of the mainstem McKenzie River above Leaburg Dam (river km 63) have included a varying proportion of hatchery‐origin spawners (pHOS) ranging from an average of 27% for the period from 2002 to 2015 (Bowerman et al., 2018), 25% for the period from 2016 to 2021 (Whitman et al., 2022), and 3% in 2022 (personal communication, J. Ziller, June 2023). Since hatchery operations began in the late 19th century, incorporation of non‐Willamette basin Chinook salmon into the McKenzie River broodstock is thought to be rare, but frequent stock transfers within the Willamette Basin likely reduced differentiation among sub‐basins (Johnson & Friesen, 2010; Myers et al., 2006). For much of the 20th century, hatchery operations on the McKenzie used a mixed broodstock consisting of Middle Fork Willamette and McKenzie stocks, but only McKenzie Basin fish have been used as broodstock since 1990 (Johnson & Friesen, 2010). The proportion of NOR Chinook salmon integrated into broodstock has varied among years, with an average of 5% between 2006 and 2012 and a current target rate of 10%–30% (Johnson & Friesen, 2014; ODFW and USACE, 2019). Tissue samples are not collected from the McKenzie River Chinook salmon broodstock and thus parentage‐based tagging is not possible in this system. Within the Willamette Basin, HOR spring Chinook salmon are most similar to NOR conspecifics from the same sub‐basin (Johnson & Friesen, 2014).

**FIGURE 1:**
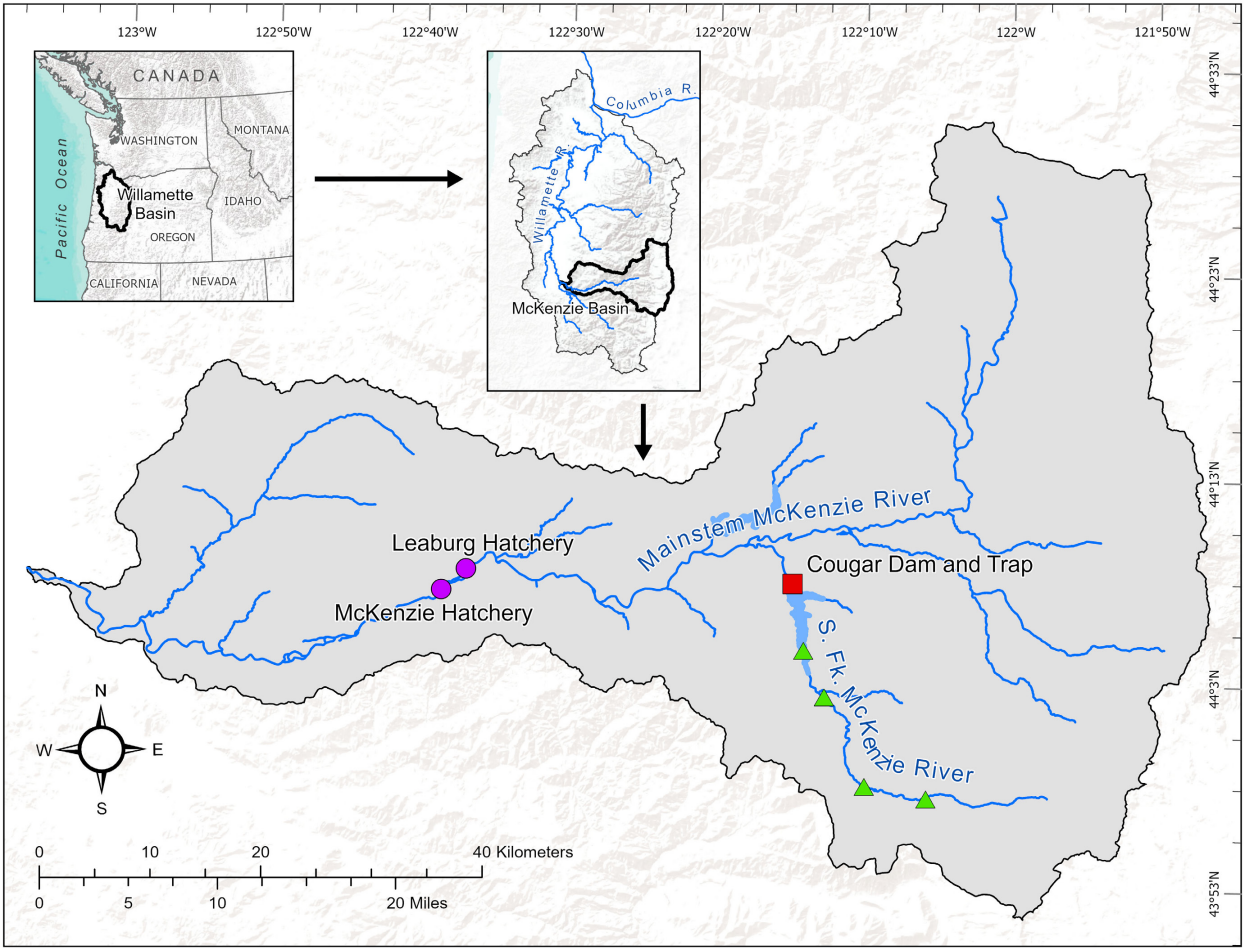
Map of McKenzie River sub‐basin including Cougar Dam (red square), McKenzie and Leaburg Hatcheries (purple circle) and release sites on South Fork McKenzie River, above Cougar Dam (green triangles).

Construction on the 158 m tall Cougar Dam was completed in 1964 on the South Fork McKenzie River. This dam blocks access to approximately 40 river km of the historically most productive reaches in the McKenzie sub‐basin (NMFS, 2008; Figure 1). Adult HOR Chinook salmon collected at the McKenzie Hatchery (river km 60) and nearby Leaburg Hatchery (river km 63) have been released above Cougar Dam (river km 103) since 1993 until the present (Figure 1). A trap‐and‐haul facility was constructed at the base of Cougar Dam in 2010 (hereafter the Cougar Trap). The Cougar Trap collects adult Chinook salmon that volitionally enter, with a relatively small percentage (~8%) of these being HOR. Importantly, wild‐born Chinook salmon collected at the Cougar Trap include offspring of individuals previously released above the dam, in addition to individuals that were produced below the dam, in the mainstem McKenzie River, or elsewhere (Banks et al., 2013, 2014, 2016; O'Malley et al., 2023; Sard et al., 2015; Sard, Johnson, et al., 2016). Wild‐born offspring of individuals previously released above the dam tends to arrive earlier at the Cougar Trap than wild‐born individuals that were not produced above the dam (Banks et al., 2014, 2016; O'Malley et al., 2023). Adfluvial females and precocial resident males also make a small contribution to the productivity of the above‐dam population (Sard, Jacobson, & Banks, 2016). Therefore, the above‐dam spawning population is composed of three sources: (1) HOR individuals collected at the McKenzie Hatchery, Leaburg Hatchery, and the Cougar Trap, (2) wild‐born individuals collected at the Cougar Trap, and (3) resident individuals above the dam.

There is no assisted downstream passage for juveniles produced above the dam. Downstream passage of outmigrants through Cougar Reservoir and Dam is associated with high mortality (Beeman et al., 2014; Duncan, 2011; Monzyk et al., 2015; Romer et al., 2016), and is likely a key factor limiting recovery (ODFW and NMFS, 2011). Productivity of the reintroduced population has been continuously evaluated since 2007 using genetic parentage analysis. These studies have found that productivity has not reached replacement in any year from 2007 to 2015 (Banks et al., 2013, 2014, 2016; O'Malley et al., 2023; Sard et al., 2015; Sard, Johnson, et al., 2016).

### 2.3 Sample collection for genetic analysis

Mass marking of HOR Chinook salmon (i.e., adipose fin removed) in the Willamette Basin began in 1997. The proportion of unmarked HOR Chinook salmon in the McKenzie River is estimated at <2% (McLaughlin et al., 2008). In the South Fork McKenzie River, 2.5% of unmarked Chinook salmon were identified as unmarked HORs (ODFW, 2017). For clarity, we consider all unmarked Chinook salmon to be wild‐born, but describe how many unmarked HORs may be present among our samples. We analyzed fin clips from nearly all wild‐born Chinook salmon that entered the Cougar Trap from 2010 to 2020 and nearly all Chinook salmon released above the dam, regardless of origin, from 2007 to 2017 (Banks et al., 2013, 2014, 2016; O'Malley et al., 2023; Sard et al., 2015; Sard, Johnson, et al., 2016). We also analyzed fin clips collected from wild‐born Chinook salmon carcasses identified during spawning ground surveys (SGSs) on the South Fork McKenzie River from 2011 to 2019, including above the dam and below the dam to the confluence with the mainstem McKenzie River. Additionally, we analyzed fin clips collected from a small number of precocial male Chinook salmon encountered during SGSs above the dam in 2014.

### 2.4 Genetic data, parentage analysis, and focal groups

We used genetic data from previous evaluations of the reintroduction effort (Banks et al., 2013, 2014, 2016; O'Malley et al., 2023; Sard et al., 2015; Sard, Johnson, et al., 2016). Methods detailing collection of genetic data and inference of the pedigree can be found in O'Malley et al. (2023). In brief, we isolated genomic DNA from fin clips, genotyped each sample at 11 microsatellite loci and a sex‐linked marker, and performed quality filtering to remove individuals genotyped at fewer than seven microsatellite loci and individuals that could have been sampled more than once. When inferring the pedigree, we defined *potential offspring* as any wild‐born individual sampled on the South Fork McKenzie, and *candidate parents* as any individual, regardless of origin, released or otherwise sampled above the dam. We assigned potential adult offspring to candidate parents using CERVUS v3.07 (Kalinowski et al., 2007) and COLONY v2.0.6.8 (Jones & Wang, 2010). We combined results from CERVUS and COLONY to generate a consensus pedigree used in all downstream analyses.

Nearly all (98%) Chinook salmon on the South Fork McKenzie express an age at maturity of 4–5 years, with approximately 2% returning at age 3 or age 6 (O'Malley et al., 2023). Given this expressed age at maturity range, and our sample collections, we were able to generate pedigrees that identified both the parents and adult offspring of candidate parents released above the dam from 2012 to 2015. Our reproductive success results are focused on candidate parents from these 4 years.

We considered three focal groups of candidate parents released above Cougar Dam from 2012 to 2015: HOR, *F*_1_ and NOR. We refer to this variable as *origin*. HORs are hatchery‐produced Chinook salmon that were reared to the smolt stage in the hatchery, released, and then collected as returning adults at the hatcheries on the mainstem or at the Cougar Trap. *F*_1_s are the first‐generation, wild‐born descendants of two HOR Chinook salmon released above the dam in previous years. Both parents of *F*_1_s must be inferred in the pedigree and both must be HOR. Finally, NORs are any wild‐born Chinook salmon released above the dam that do not assign to a parent previously released above the dam. These individuals are presumed to have been produced below the dam, on the mainstem McKenzie River, or elsewhere. NORs possess an unknown level of hatchery ancestry. We did not consider other candidate parents from 2012 to 2015 that do not fall into one of these three groups. For example, individuals that were assigned to a single parent, or individuals that were offspring of mixed (i.e., HOR × NOR) mate pairs were not analyzed, due to low sample size.

### 2.5 Body length

Body length is positively associated with lifetime reproductive success in salmonids (Koch & Narum, 2021). We compared body lengths of HORs, *F*_1_s, and NORs released above the dam from 2012 to 2015 with body lengths of wild‐born individuals encountered as carcasses on the mainstem McKenzie River, and on the South Fork McKenzie River below Cougar Dam from 2012 to 2015. Body length was measured for HORs, *F*_1_s, and NORs at the Cougar Trap prior to release above the dam. Body length was measured for carcass samples (*n* = 610) during spawning ground surveys conducted on the mainstem and South Fork McKenzie River. We fit a linear model on body length with three fixed effects, *year*, *sex*, and *group* (HOR, *F*_1_, NOR, Carcass), and the *group × sex* interaction. We validated the model using Pearson residuals and evaluated significance with an *F*‐test and type II sums of squares. We used the *emmeans* package in R to conduct post‐hoc estimation of marginal mean lengths and hypothesis testing.

### 2.6 Relative reproductive success

We defined total lifetime fitness (TLF) as the number of adult offspring produced by each candidate parent released above the dam (O'Malley et al., 2023). To address if there were fitness differences among HORs, *F*_1_s and NORs, we first estimated relative reproductive success (RRS) using a model‐based approach that permits parsing of the effect of *origin* from covariates such as *body length* and *year*. In a complementary approach, we estimated RRS by calculating simple ratios of mean TLF between all pairwise comparisons of HORs, *F*_1_s, and NORs. To distinguish between the two approaches, we refer to RRS estimates as _model_RRS and _Δ_RRS, respectively.

In the model‐based approach, we began by fitting a generalized linear mixed model on TLF using the *glmmTMB* package in *R*. In addition to the effect of *origin* on TLF, we considered the influence of multiple potential covariates including *sex*, *body length*, *transport day*, and *year* and two interaction terms, *sex × transport day* and *sex × origin*. We also included *transport group* as a random effect. *Transport day* was the Julian calendar day that individuals were released and was modeled as a continuous fixed effect. Previous work suggested that non‐linear effects for *transport day* such as disruptive or stabilizing selection were not necessary, and there was insufficient evidence to support a *year × transport day* interaction (O'Malley et al., 2023). *Transport group* was the set of individuals released at a single location during a single day. Our modeling followed the recommendations of Zuur et al. (2009) and Bolker (2015). We conducted an exploratory data analysis to understand the relationship among predictors and compared model fit under negative binomial and zero‐inflated negative binomial distributions. Model fit under different distributions was evaluated using AIC, BIC, rootograms and QQ‐plots of randomized quantile residuals from the *COUNTREG* package in *R*. To quantify multicollinearity among categorical and continuous predictors we used the generalized variance inflation factor (GVIF^1/(2 × df)^) (Fox & Monette, 1992), and a conservative cutoff of 2.0 (see Appendix S2, section TLF Variation Model, for additional details).

After exploratory data analysis, we fit models using the negative binomial distribution and a log link function. For model selection, we first identified the best random effects structure by fitting a fully saturated fixed effect model using restricted maximum likelihood and varying the random effects. Model selection of random effects was by AIC. After refitting the fully saturated fixed effects model with the final random effects structure using maximum likelihood, we conducted backwards stepwise model selection for fixed effects based on likelihood ratio tests for each predictor, and a *p*‐value cutoff of 0.05. We also considered alternative models with marginal differences in support from the final model (ΔAIC < 2). Once a final model was selected (hereafter GLMM_TLF_), we conducted model validation by testing for goodness of fit, overdispersion, and influence of outliers using residuals simulated by the *DHARMa* package in *R*. Effect plots of significant predictors retained in the GLMM_TLF_ were generated using the *effects* package in *R* and conditioned on the typical values of all other significant predictors in the final model. We used the *emmeans* package in R to conduct post‐hoc estimation of _model_RRS and hypothesis testing. We estimated _model_RRS by contrasting the marginal mean fitness for each level of *origin* after controlling for other significant predictors in the final model. We used cell‐based weighting during estimation of marginal means to accommodate the unbalanced sample sizes across *origin* and *year*. We considered _model_RRS to be significantly different than one if the Tukey adjusted *p*‐values for these post‐hoc contrasts were less than 0.05.

In the second approach to determine if RRS was different than one, we defined _Δ_RRS as the ratio between mean TLF of all pairwise comparisons of HORs, *F*_1_s and NORs, within years. We conducted an additional analysis within both years and sexes. Confidence intervals for _Δ_RRS were estimated using a maximum likelihood approach following Kalinowski and Taper (2005). _Δ_RRS was considered different from one if the confidence interval did not include one.

### 2.7 Adult offspring characteristics

We also examined if the adult offspring produced by HORs, *F*_1_s, and NORs differed in age at maturity or body length. We chose to model offspring age using a binomial generalized linear model with the proportion of age‐5 offspring versus age‐4 offspring as the response variable, because nearly all (98%) spring Chinook salmon in the McKenzie River return at either age 4 or at age 5. We used three fixed explanatory variables: *origin* of parents, *year* of parents, and *sex*. We also included the interaction *origin × sex*. Model validation, effect plotting and post‐hoc analysis followed the approach used with GLMM_TLF_. We examined adult offspring body length using the same fixed effects structure, but a linear model. We validated the model using Pearson residuals and evaluated significance with an *F*‐test and type II sums of squares.

## 3 RESULTS

### 3.1 Parentage and focal group sample size

Our pedigree included 9839 individuals sampled from 2007 to 2020 (O'Malley et al., 2023). Of these individuals, 2027 HOR Chinook salmon and 952 wild‐born Chinook salmon were released, or sampled above the dam during 2012–2015. Among the 952 wild‐born Chinook salmon released, or sampled above the dam during this period, 15 individuals without body length measurements and 235 individuals that were assigned to a single parent or mixed mate pair were excluded from further analysis. Of the remaining individuals, 465 were identified as *F*_1_s and 237 were identified as NORs (Table 1, Table S1). Note that due to incomplete marking of HOR Chinook salmon (2.0% mis‐clip in McKenzie basin and, 2.5% mis‐clip rate in South Fork McKenzie) (McLaughlin et al., 2008; ODFW, 2017), approximately 19–24 of the 952 unmarked individuals in our study are likely HORs. Since they will not be assigned to a parent and are unmarked, these individuals will be misidentified as NORs (~8%–10% of NORs).

**TABLE 1:** Number of candidate parents by origin and year collected from 2012 to 2015 released above Cougar Dam.

Year	HOR	*F*_1_	NOR
2012	446	275	174
2013	454	127	26
2014	506	48	25
2015	619	15	12

*Note*: HOR are any hatchery‐produced Chinook salmon collected at either the McKenzie or Leaburg Hatchery or Cougar Trap. *F*_1_s are first‐generation, wild‐born descendants of two HOR parents that were released above the dam in previous years. NOR are wild‐born Chinook salmon that do not assign to a parent previously released above the dam and are presumed to be produced below the dam, on the mainstem, or elsewhere. Wild‐born Chinook salmon released above the dam beginning in 2010. Modifications to trap‐and‐haul operations reduced the number of wild‐born Chinook salmon released above the dam in 2013 and subsequent years (O'Malley et al., 2023; Sard et al., 2015).

### 3.2 Body length

Body length was significantly explained by *year*, and the *sex × group* interaction (*F*‐test *p*‐value <2e‐16, type II sums of squares). In our post‐hoc analyses, we conditioned marginal mean length on *sex* and averaged across all levels of *year*, and therefore present results separately for males and females. For females, only pairwise contrasts that included HORs were significant. HOR females were smaller than every other group of females (Figure S1, Tukey *p*‐value < 0.001). For males, every pairwise contrast was significant (Tukey *p*‐value < 0.01). HOR males had the shortest body length, followed by *F*_1_s, NORs, and finally, carcasses (Figure S1).

### 3.3 Relative reproductive success

Several predictors used in the generalized linear mixed model of TLF were correlated with *origin*. HOR individuals were smaller on average and included more females than both *F*_1_s and NORs (Figure S1 and Table S2, also see Section 2.5 above). *F*_1_s tended to be released above the dam earlier than both HORs and NORs (Figure S2). However, we did not find evidence of strong multicollinearity among the predictors as assessed with the generalized variance inflation factor, indicating that despite the relationships between predictors, there is sufficient information in the dataset to parse their individual effects.

**FIGURE 2:**
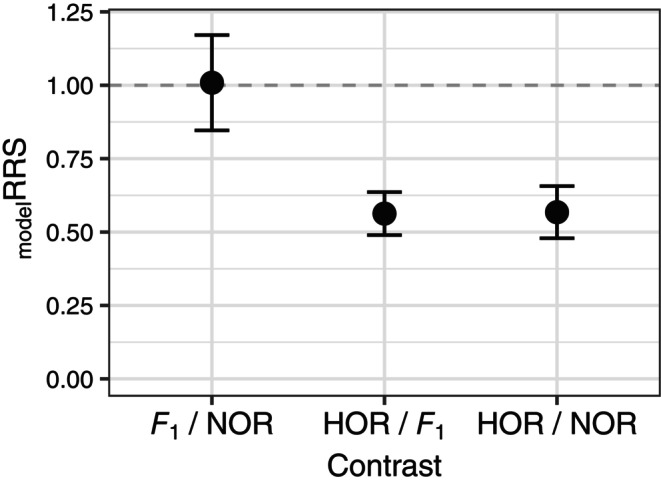
_model_RRS. Post‐hoc contrasts of marginal mean total lifetime fitness between different levels of *origin* in GLMM_TLF_ after controlling for effects of *body length* and *year* (_model_RRS). Error bars are the standard error of the _model_RRS estimate. Horizontal line at _model_RRS = 1 indicated equal fitness between the two groups. Levels of *origin* are defined as HOR, *F*_1_, and NOR. HOR are any hatchery‐produced Chinook salmon collected at either the McKenzie or Leaburg Hatchery or Cougar Trap. *F*_1_s are first‐generation, wild‐born descendants of two HOR parents that were released above the dam in previous years. NOR are wild‐born Chinook salmon that do not assign to a parent previously released above the dam and are presumed to be produced below the dam, on the mainstem, or elsewhere.

After model selection, the final model (GLMM_TLF_) included *origin*, *length*, and *year* as fixed predictors and *transport group* as a random effect (see Appendix S2 for additional details). We did not find that *sex* or a *sex × origin* interaction improved the model fit to the data and therefore present _model_RRS for both sexes together. Parameter estimates and standard errors, as well as significance testing for each predictor retained in GLMM_TLF_, are presented in Table 2. Predicted *F*_1_ and NOR fitness was greater than HOR fitness (Wald‐test *p*‐value 7.75e–5 and 9.85e–5, respectively, Table 2, Figure S3). _model_RRS for HOR versus *F*_1_ and for HOR versus NOR contrasts were significantly different than one (_model_RRS = 0.56 and 0.57 respectively, Figure 2, Table 3). _model_RRS for the *F*_1_ versus NOR contrast was not significantly different than one (Figure 2, Table 3).

**TABLE 2:** GLMM_TLF_ model fit. Generalized linear mixed model examining the influence of *origin*, *body length*, *year*, on total lifetime fitness (TLF).

**Fixed effects**	***β***	**SE**	**LRT *p*‐value**	**Wald *p*‐value**
Intercept	−6.942	0.590		
Origin	0.527	0.133	**4.3e–05**	**7.8e–05**
Origin [NOR]	0.635	0.163		**9.9e–05**
Body length	0.067	0.007	**5.1e–21**	**2.0e–16**
Year [2013]	0.708	0.141	**3.6e–6**	**5.4e–07**
Year [2014]	−0.028	0.161		0.86
Year [2015]	−0.033	0.184		0.86

**Random effects**	***σ*** ^**2**^	**SD**		
Release group	0.040	0.200		

*Note*: The full set of predictors explored during model selection included *origin*, *sex*, *body length*, *release day*, *year*, *sex × origin*, and *release day × origin*. *Release group* was included as random effect. Estimated effect (*β*) and standard error (SE) of each fixed predictor on the link scale (log) for predictors that were retained in the final model are presented. The null hypothesis that each predictor did not significantly improve the model fit was tested with a likelihood ratio test (LRT *p*‐value). The null hypothesis that each predictor has an effect significantly different from zero for continuous predictors and different from the focal level for categorical variables was evaluated with the Wald test (Wald *p*‐value). Focal level for *origin* was HOR, and *year* was 2012. Estimated variance (*σ* ^2^) and standard deviation (SD) are presented for random effects. Significant *p*‐values are in bold. Levels of *origin* are defined as HOR, *F*_1_, and NOR. HOR are any hatchery‐produced Chinook salmon collected at either the McKenzie or Leaburg Hatchery or Cougar Trap. *F*_1_s are first‐generation, wild‐born descendants of two HOR parents that were released above the dam in previous years. NOR are wild‐born Chinook salmon that do not assign to a parent previously released above the dam and are presumed to be produced below the dam, on the mainstem, or elsewhere.

**TABLE 3:** _model_RRS.

Contrast	_model_RRS	SE	*p*‐Value
HOR/*F*_1_	0.563	0.073	<0.0001
HOR/NOR	0.568	0.089	0.001
*F*_1_/NOR	1.009	0.162	0.999

*Note*: Post‐hoc contrasts of marginal mean total lifetime fitness between different levels of *origin* in GLMM_TLF_ after controlling for effects of *body length* and *year* (_model_RRS). SE. is the standard error of the _model_RRS estimate. *p*‐Values are Tukey‐adjusted for three pairwise comparisons. Levels of *origin* are defined as HOR, *F*_1_, and NOR. HOR are any hatchery‐produced Chinook salmon collected at either the McKenzie or Leaburg Hatchery or Cougar Trap. *F*_1_s are first‐generation, wild‐born descendants of two HOR parents that were released above the dam in previous years. NOR are wild‐born Chinook salmon that do not assign to a parent previously released above the dam and are presumed to be produced below the dam, on the mainstem, or elsewhere.

Relative to GLMM_TLF_, a model that also included the effect of *transport day* marginally improved the fit to the data (ΔAIC = 1.8, likelihood ratio test *p*‐value = 0.053). Because *transport day* varies across the levels of *origin*, and potentially influences our estimate of _model_RRS, we also considered this model and have provided the model fit and post‐hoc analysis as supplements (Tables S3 and S4). We note that both parameter estimates and post‐hoc significance testing were qualitatively similar to those from GLMM_TLF_.

**FIGURE 3:**
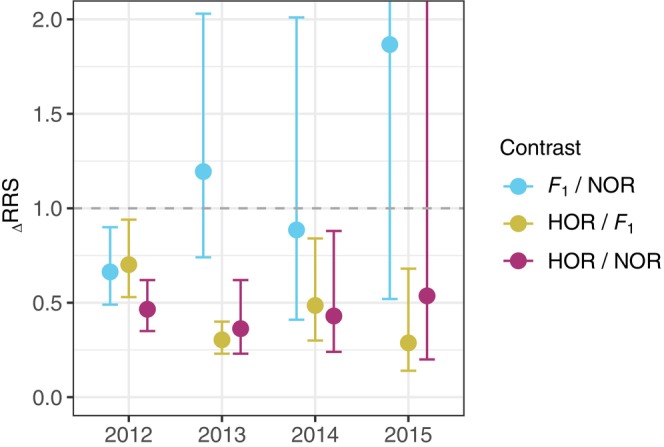
_Δ_RRS. Ratio between mean total lifetime fitness between different groups (_Δ_RRS). Error bars are maximum likelihood‐based 95% confidence intervals of _Δ_RRS. The confidence interval for the *F*_1_ versus NOR contrast extends beyond the plot extent. HOR are any hatchery‐produced Chinook salmon collected at either the McKenzie or Leaburg Hatchery or Cougar Trap. *F*_1_s are first‐generation, wild‐born descendants of two HOR parents that were released above the dam in previous years. NOR are wild‐born Chinook salmon that do not assign to a parent previously released above the dam and are presumed to be produced below the dam, on the mainstem, or elsewhere.

We found similar patterns for _Δ_RRS to _model_RRS for both HOR versus *F*_1_ and HOR versus NOR contrasts. _Δ_RRS for the HOR versus *F*_1_ contrast (_Δ_RRS = TLF_HOR_/TLF_*F*1_) was less than one in all years evaluated (Figure 3). _Δ_RRS for the HOR versus NOR contrast (_Δ_RRS = TLF_HOR_/TLF_NOR_) was significantly less than one in three of the 4 years (Figure 3). Similar to our model‐based approach, we did not find strong differences in these _Δ_RRS contrasts between sexes, but _Δ_RRS was significantly less than one in fewer years for males than females. Specifically, when we split the data by sex, _Δ_RRS for both HOR versus *F*_1_ and HOR versus NOR contrasts were significantly less than one for males in 2 of 4 years, and for females in 3 of 4 years (Figure S4). There was one notable difference between _Δ_RRS and _model_RRS results: _Δ_RRS for the *F*_1_ versus NOR contrast (_Δ_RRS = TLF_*F*1_/TLF_NOR_) was significantly less than one in 2012 (Figure 3). When we split the data by sex, we found _Δ_RRS for this contrast was less than one in 2012 for males, but not females (Figure S4).

### 3.4 Adult offspring characteristics

**FIGURE 4:**
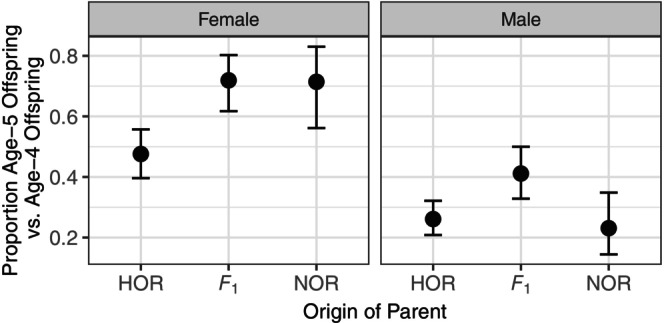
Estimated proportion of age‐5 versus age‐4 female (left panel) and male (right panel) offspring produced by HORs, *F*_1_s, and NORs. Error bars are 95% confidence limits of the estimates. Levels of *origin* are defined as HOR, *F*_1_, and NOR. HOR are any hatchery‐produced Chinook salmon collected at either the McKenzie or Leaburg Hatchery or Cougar Trap. *F*_1_s are first‐generation, wild‐born descendants of two HOR parents that were released above the dam in previous years. NOR are wild‐born Chinook salmon that do not assign to a parent previously released above the dam and are presumed to be produced below the dam, on the mainstem, or elsewhere.

Model fit to the proportion of *age‐5* versus *age‐4* offspring produced by candidate parents released above the dam was not significantly improved by including *year* of parents or the interaction between *year* and *origin* (ΔAIC <1, likelihood ratio test *p*‐value >0.05), so the data were pooled across years. Parameter estimates and standard errors, as well as significance testing for the effects of *origin* of parents, *sex* of offspring, and their interaction on the age at maturity of their offspring are presented in Table 4. Female adult offspring of *F*_1_s and NORs returned at an older age than female adult offspring of HORs (Figure 4). Male adult offspring of *F*_1_s returned at an older age than male adult offspring of both HORs and NORs (Figure 4). We found similar trends in body length between the levels of *origin* and *sex*, but only one post‐hoc contrast was significant, possibly due to a smaller number of individuals with length measurements than age at maturity estimates (Table S5).

**TABLE 4:** Offspring age at maturity model fit.

Predictor	*β*	SE	LRT *p*‐value	Wald *p*‐value
Intercept	−0.097	0.166		
Origin	1.037	0.289		**3.3e–04**
Origin [NOR]	1.013	0.380		**7.8e–03**
Sex [Male]	−0.945	0.224		**2.5e–05**
Generation × Sex [*F*_1_ × Male]	−0.354	0.373	**0.05**	0.34
Generation × Sex [NOR × Male]	−1.175	0.504		**0.02**

*Note*: Binomial generalized linear model examining the influence of *origin*, *sex*, and their interaction on proportion of age‐5 versus age‐4 adult offspring produced by candidate parents. Estimated effect (*β*) and standard error (SE) of each fixed predictor on the link scale (log odds ratio) for predictors that were retained in the final model are presented. The null hypothesis that each predictor did not significantly improve the model fit was tested with a likelihood ratio test (LRT *p*‐value). The null hypothesis that each predictor has an effect significantly different from the focal level of the predictor was evaluated with the Wald test (Wald *p*‐value). Focal level for *origin* is HOR, and sex is female. Significant *p*‐values are in bold. Levels of *origin* are defined as HOR, *F*_1_, and NOR. HOR are any hatchery‐produced Chinook salmon collected at either the McKenzie or Leaburg Hatchery or Cougar Trap. *F*_1_s are first‐generation, wild‐born descendants of two HOR parents that were released above the dam in previous years. NOR are wild‐born Chinook salmon that do not assign to a parent previously released above the dam and are presumed to be produced below the dam, on the mainstem, or elsewhere.

## 4 DISCUSSION

### 4.1 Relative reproductive success

Low relative reproductive success of HOR Pacific salmonids challenges reintroduction and supplementation programs and prompts an important question: does lower fitness of HOR individuals in the wild persist among their wild‐born descendants? We found that *F*_1_s (i.e., first‐generation, wild‐born descendants of two HOR Chinook salmon) produced significantly more adult offspring than HOR Chinook salmon that spawned alongside them in the same river. The strongest evidence of this generational fitness advantage comes from our model‐based estimates of RRS, which reduce bias by accounting for interannual variation and for potential fitness covariates such as sex, release strategies, and body length. However, this finding is also supported by directly contrasting mean fitness between *F*_1_s and HORs in each year.

To our knowledge, only one other study has explicitly addressed this question in Chinook salmon (Nuetzel et al., 2023), and no study has compared the fitness of wild‐born hatchery descendants to other wild‐born Chinook salmon spawning alongside them in the same river. We found only weak evidence of fitness differences between *F*_1_s and NORs (i.e., wild‐born individuals of unknown parentage), with direct contrasts of mean TLF suggesting that NORs produced more adult offspring than *F*_1_s in only one (2012) of 4 years. Importantly, this year had the highest sample size, suggesting that there may be limited power to identify significant differences in mean fitness in the other 3 years using this method. However, by not accounting for increased fitness among *F*_1_s associated with *body length*, these direct contrasts may overestimate fitness differences directly attributable to *origin*. Our model‐based estimates suggest that *F*_1_s and NORs produce equal numbers of adult offspring after accounting for covariates. We conclude that fitness differences between *F*_1_s and NORs, if present, are minor relative to the differences between either group and HORs. Our results corroborate the previous observation that *F*_1_s produced more juvenile offspring (age 0) than HORs, but a similar number of juvenile offspring to NORs in 2012 and 2013 (Banks et al., 2016). Together, our two central findings that *F*_1_ fitness is greater than HOR fitness but not greater than NOR fitness suggest that a single generation in the wild increases fitness for descendants of HOR Chinook salmon, such that they may be comparable to wild‐born Chinook salmon that might naturally colonize available habitat through dispersal if the passage barrier was not present. Our findings offer support for the continued use of HOR Chinook salmon to initially reestablish naturally spawning populations in habitats where they have been extirpated (but see Section 4.3 below).

### 4.2 Adult offspring characteristics

In addition to evaluating the RS of the three groups of adult Chinook salmon released above the dam, we also considered two ecologically relevant phenotypes of their adult offspring, age at maturity, and body length. We found that female adult offspring of *F*_1_s and NORs returned as adults at an older age than female adult offspring of HORs, whereas male adult offspring of *F*_1_s returned as adults at an older age than male adult offspring of both HORs and NORs. We found similar trends in body length, which is consistent with the strong correlation between these two traits (Reed et al., 2019). One interpretation of these patterns is that while there were differences in age and size at maturity of adult offspring between sexes, these sex differences were weakest among offspring of HORs and *F*_1_s, and greatest among offspring of NORs. This framing prompts two questions, why might there be less age and size variation across sexes within Chinook salmon with recent, known hatchery ancestry, and what are the evolutionary and conservation implications of this reduced variation?

Age at maturity and fitness are positively associated with salmonids (Ohlberger et al., 2020). The observation that *F*_1_s produced older offspring than HOR Chinook salmon across both sexes suggests that subsequent generations of wild‐born hatchery descendants (e.g., *F*_2_s) may have greater fitness. However, age differences between the adult offspring of *F*_1_s and NORs depended on offspring sex. Trade‐offs between survival to maturity and size or age at maturity are commonly observed among salmonids, and result in sexually antagonistic selection, with early maturation favored in males more so than in females (Berejikian et al., 2010; Seitz et al., 2019). This sexually antagonistic selection may be responsible for the maintenance of diversity in age at maturity, and therefore increased fishery and population resilience (Greene et al., 2010; Schindler et al., 2010). Sexually antagonistic selection for age at maturity may be resolved in Chinook salmon via sex‐specific haplotypes (McKinney et al., 2021). In Chinook salmon, the effects of male‐specific age at maturity associated haplotypes may be reduced in the hatchery relative to the natural environment (McKinney et al., 2021), and this conditional neutrality may lead to evolved changes in the genetic architecture of this trait among hatchery descendants (Van Dyken & Wade, 2010). Our finding of reduced sex dependence in age at maturity phenotypes among offspring of HORs and their descendants, relative to NORs, warrants a note of caution about ignoring the crucial role of diversity for long‐term species persistence. Specifically, hatchery descendants may achieve fitness similar to that of wild Pacific salmonids, but may lack adequate genetic and phenotypic diversity to adapt to or recover from short‐term disturbance. Ultimately, we do not know if there is reduced genetic variation in age at maturity associated genetic loci among hatchery descendants, and further investigation is required to address this concern.

### 4.3 Limitations

There are important limitations to our findings that must be considered before applying our conclusions to other reintroductions, hatchery supplementation programs, hatchery augmentation programs, or hatchery risk evaluations, in general.

The first limitation is related to the reciprocal influences of integrating wild‐born individuals into the hatchery broodstock, and interbreeding between HOR and wild‐born individuals in the wild. Low RRS of HOR Chinook salmon in the wild may be ameliorated by integrating local‐origin, wild‐born individuals into a hatchery broodstock (Hess et al., 2012; Janowitz‐Koch et al., 2019; Waters et al., 2015, 2018), but see Koch et al. (2022). It is possible that sustained natural production and limited non‐local origin stock transfers in the Upper Willamette Basin, coupled with integration of the McKenzie Hatchery broodstock and other hatchery practices (e.g., random selection of individuals for broodstock, brood collected throughout the run period, number of breeders per cohort, etc.), have maintained adaptive genetic diversity and the capacity for increased fitness among the wild‐born descendants of HORs. Similar findings by Nuetzel et al. (2023) also stem from early generation, wild‐born descendants of HOR Chinook salmon from an integrated broodstock. Therefore, our finding that a single generation in the wild increases fitness among hatchery descendants may not be applicable to non‐local origin or segregated broodstocks. Reciprocally, interbreeding between hatchery and wild‐born Pacific salmonids in the wild may reduce genetic diversity and fitness of natural populations (Baskett & Waples, 2013; Ford, 2002; Ryman & Laikre, 1991; Willoughby & Christie, 2019). Our finding that *F*_1_ fitness is comparable to that of NORs is potentially mediated by the extent to which hatchery production has historically influenced the natural population in the McKenzie River. In the period from 2002 to 2015, pHOS averaged 27% in the McKenzie River (Bowerman et al., 2018), suggesting that under random mating and equal fitness of offspring, 7.3% (i.e., 0.27 × 0.27) of NORs may be descendants of two HOR parents that spawned below Cougar Dam or elsewhere. We expect this estimate to be an upper limit given the lower reproductive success of HOR relative to wild‐born Chinook salmon. However, it serves as an important reminder that all NORs may have some degree of hatchery ancestry. Fitness differences between *F*_1_s and NORs may be greater in populations with less hatchery influence on the natural population.

A second limitation that must be considered before generalizing our results to other systems is that there is no natural, locally adapted above‐dam population against which to make fitness comparisons. We should exercise caution before considering NORs as proxies for individuals from such a population, as the spatial scale of local adaptation is unknown. NORs released above the dam must volitionally enter the Cougar Trap. NORs tend to arrive at the Cougar Trap later than individuals produced above Cougar Dam, including *F*_1_s (Banks et al., 2014, 2016; O'Malley et al., 2023). While this difference may reflect heritable variation in migration timing between *F*_1_s and individuals from the natural McKenzie River population, it is also possible that NORs represent late‐season dispersers from below the dam, or strays from another river. Studies of sockeye (*O. nerka*) and Atlantic salmon suggest that late‐season dispersers previously homed to their natal habitat (Peterson et al., 2016), and have lower fitness than successfully homing individuals in the same environment (Mobley et al., 2019; Peterson et al., 2014). We also found that NOR males were smaller than other wild‐born Chinook salmon encountered during spawning ground surveys below the dam and on the mainstem. Body length is positively associated with fitness in salmonids (Koch & Narum, 2021), a relationship confirmed by our modeling. Therefore, while *F*_1_ fitness was substantially greater than HOR fitness and similar to NOR fitness, NOR fitness may not represent that reached by locally adapted individuals returning to their natal habitat to spawn. Size differences between NOR males and carcasses sampled below the dam might also be explained by size‐dependent observation errors that can occur during spawning ground surveys (Zhou, 2011). The possibility that mean fitness of NORs in our study could differ from a hypothetical natural and locally adapted population is particularly important in a supplementation context.

However, in a reintroduction context, there are no locally adapted individuals against which to contrast *F*_1_ fitness. Pacific salmonid reintroduction programs generally seek to reestablish highly productive, self‐sustaining populations, but face substantial uncertainty (Anderson et al., 2014; Lusardi & Moyle, 2017; McClure et al., 2018). Therefore, managers must choose between seeding a reintroduction with hatchery individuals, wild‐born individuals collected from natural populations, volitional dispersers, or a mix to balance risks to extant natural populations with the goal of maximizing productivity and growth rate of the reintroduced population. Contrasts between *F*_1_ and NOR fitness allow evaluation of alternative reintroduction management strategies, namely, the choice between using hatchery individuals and their descendants, or relying on natural colonization. Absence of fitness differences between *F*_1_s and NORs in the McKenzie River suggests that descendants of hatchery Chinook salmon may be as productive as non‐local, volitional dispersers allowed to naturally colonize vacant habitats.

Finally, we caution that our findings do not negate prior evidence that has demonstrated low RRS of HOR salmonids (Christie et al., 2014; Koch & Narum, 2021), and the risk that chronically elevated hatchery influence can pose to the genetic integrity and productivity of natural populations (McMillan et al., 2023; Willoughby & Christie, 2019). This caution is particularly salient to harvest augmentation programs intended to increase fishing and harvest opportunities without impairing naturally reproducing populations (ODFW, 2010). While our results offer hope that naturally spawning Chinook salmon populations can be established with HOR individuals and reestablished populations may experience generational increases in mean fitness, they also demonstrate the relatively low parental contribution to population productivity from HOR individuals spawning in the wild. This limited contribution to productivity should be carefully weighed against the potential risks HOR Chinook salmon pose to the fitness of NOR spawners. Repeated interbreeding between HOR and NOR individuals may result in a decline in mean fitness of NOR Chinook salmon unless the level of interbreeding is managed to a low level.

## 5 CONCLUSION AND FUTURE DIRECTIONS

We found that the fitness of hatchery descendants (*F*_1_s) increases after a single generation in the wild, such that the fitness of *F*_1_s is indistinguishable from that of other wild‐born Chinook salmon released into the same habitat to spawn. We also found that a trait positively associated with fitness, age at maturity, is increased among second‐ relative to first‐generation hatchery descendants, suggesting that fitness increases may continue in subsequent generations. However, our findings likely depend on the pattern of gene flow between the hatchery broodstock and the natural population in the McKenzie River and care must be taken before generalizing to other populations.

Our data do not identify the mechanistic basis of the observed differences in fitness. If reduced reproductive success among hatchery salmonids is driven by domestication selection (Christie et al., 2012, 2016; Waters et al., 2018), and domestication has severely reduced genetic variance for traits under selection in the wild, the pace of adaptation to natural conditions may be too slow to be ecologically important on conservation‐relevant timescales. Our findings suggest that either low RRS is driven by phenotypic plasticity in response to the hatchery environment (e.g., growth, behavior, etc.), or that despite domestication selection, improved hatchery practices (e.g., the proportion of wild‐born individuals into broodstock, the effective number of breeders per cohort, etc.) may maintain sufficient genetic variance on which selection to natural conditions can act, and adaptation is rapid. Because we cannot conclude whether the increase in fitness between *F*_1_s and HORs is due to genetic changes or environmental influences on phenotypic plasticity, we encourage continued research into the mechanisms that drive low RRS of HOR Pacific salmonids.

## FUNDING INFORMATION

This work was funded by the US Army Corps of Engineers.

## CONFLICT OF INTEREST STATEMENT

The authors declare no conflicts of interest.

## Supporting information


Appendix S1.

Appendix S2.

## Data Availability

Detailed logs and all data from this study are available at a github repository, https://github.com/david‐dayan/mckenzie_naturalization, and archived at zenodo with a stable identifier (DOI: 10.5281/zenodo.8097487). Raw data will be archived at the Dryad Digital Repository following acceptance for publication.
