# Supplementary material for: A single generation in the wild increases fitness for descendants of hatchery‐origin Chinook salmon (Oncorhynchus tshawytscha)
Source: Evol Appl. 2024 Apr 11;17(4):e13678. doi: 10.1111/eva.13678 (PMC11009425; doi:10.1111/eva.13678)
Supplement: Supplementary file 1 — Appendix S1. [file EVA-17-e13678-s002.docx]

# Supplemental Tables

**Supplemental Table 1** Detailed number of candidate parents by group collected from 2012 – 2015 released above Cougar Dam including groups that were excluded due to low sample size (W_HxW_) or clarity (Single Parent). Groups excluded from the analysis are marked with an asterisk. HOR are any hatchery-produced Chinook salmon collected at either the McKenzie or Leaburg Hatchery or Cougar Trap. F_1_s are first-generation, wild-born descendants of two HOR parents that were released above the dam in previous years. NOR are wild-born Chinook salmon that do not assign to a parent previously released above the dam and are presumed to be produced below the dam, on the mainstem, or elsewhere. W_HxW_ and W_WxH_ are wild-born Chinook salmon descended from mixed mate pairs (Dam x Sire). Single parent refers to Chinook salmon that were assigned to only one parents. F_2_ refers to wild-born descendants of two wild-born parents that were released above the dam in previous years.

| **Year** | **Sample Location** | **HOR** | **F_1_** | **NOR** | ***W_HxW_** | ***W_WxH_** | ***Single Parent** | ***F_2_** |
| --- | --- | --- | --- | --- | --- | --- | --- | --- |
| 2012 | Hatchery | 429 |  |  |  |  |  |  |
|  | Cougar Trap or above | 17 | 275 | 174 |  |  | 50 |  |
| 2013 | Hatchery | 439 |  |  |  |  |  |  |
|  | Cougar Trap or above | 15 | 127 | 26 |  | 1 | 18 |  |
| 2014 | Hatchery | 486 |  |  |  |  |  |  |
|  | Cougar Trap or above | 20 | 48 | 25 | 20 | 2 | 29 | 8 |
| 2015 | Hatchery | 600 |  |  |  |  |  |  |
|  | Cougar Trap or above | 19 | 15 | 12 | 37 | 9 | 39 | 22 |

**Supplemental Table 2** Sex ratios by origin. HOR are any hatchery-produced Chinook salmon collected at either the McKenzie or Leaburg Hatchery or Cougar Trap. F_1_s are first-generation, wild-born descendants of two HOR parents that were released above the dam in previous years. NOR are wild-born Chinook salmon that do not assign to a parent previously released above the dam and are presumed to be produced below the dam, on the mainstem, or elsewhere.

| **Origin** | **n_male_** | **n_female_** | **proportion female** |
| --- | --- | --- | --- |
| HOR | 771 | 1254 | 0.62 |
| F_1_ | 278 | 187 | 0.40 |
| NOR | 153 | 84 | 0.35 |

**Supplemental Table 3** GLMM model fit with final predictors from GLMM_TLF_ and *transport day*. Response variable was total lifetime fitness (TLF). Estimated effect (β) and standard error (s.e.) of each fixed predictor on the link scale (log) for predictors. The null hypothesis that each predictor did not significantly improve the model fit was tested with a likelihood ratio test (LRT p-value). The null hypothesis that each predictor has an effect significantly different from zero for continuous predictors and different from the focal level for categorical variables was evaluated with the Wald test (Wald p-value). Focal level for *origin* was HOR, and *year* was 2012. Estimated variance (σ^2^) and standard deviation (s.d.) are presented for random effects. Significant p-values are in bold. Levels of *origin* are defined as HOR, F_1_, and NOR. HOR are any hatchery-produced Chinook salmon collected at either the McKenzie or Leaburg Hatchery or Cougar Trap. F_1_s are first-generation, wild-born descendants of two HOR parents that were released above the dam in previous years. NOR are wild-born Chinook salmon that do not assign to a parent previously released above the dam and are presumed to be produced below the dam, on the mainstem, or elsewhere.

| **Fixed Effects** | **β** | **s.e.** | **LRT p-value** | **Wald p-value** |
| --- | --- | --- | --- | --- |
| Intercept | -6.846 | 0.589 |  |  |
|  |  |  |  |  |
| Origin [F_1_] | 0.363 | 0.158 | **1.7e-03** | **2.2e-02** |
| Origin [NOR] | 0.593 | 0.165 |  | **3.2e-04** |
|  |  |  |  |  |
| Body Length | 0.067 | 0.007 | **2.2e-16** | **2.0e-16** |
|  |  |  |  |  |
| Transport Day | -0.004 | 0.002 | 5.2e-02 | 5.5e-02 |
|  |  |  |  |  |
| Year [2013] | 0.688 | 0.142 | **8.7e-06** | **1.3e-06** |
| Year [2014] | -0.040 | 0.161 |  | 8.0e-01 |
| Year [2015] | -0.099 | 0.188 |  | 6.0e-01 |
|  |  |  |  |  |
| **Random Effects** | **σ^2^** | **s.d.** |  |  |
| Transport Group | 0.041 | 0.201 |  |  |

**Supplemental Table 4** _model_RRS from GLMM with *transport day*. *Post-hoc* contrasts of marginal mean total lifetime fitness between different levels of *origin* after controlling for effects of *release day, body length* and *year*. s.e. is the standard error of the _model_RRS estimate. p-values are Tukey-adjusted for three pairwise comparisons. Levels of *origin* are defined as HOR, F_1_, and NOR. HOR are any hatchery-produced Chinook salmon collected at either the McKenzie or Leaburg Hatchery or Cougar Trap. F_1_s are first-generation, wild-born descendants of two HOR parents that were released above the dam in previous years. NOR are wild-born Chinook salmon that do not assign to a parent previously released above the dam and are presumed to be produced below the dam, on the mainstem, or elsewhere.

| **Contrast** | **_model_RRS** | **s.e.** | **p-value** |
| --- | --- | --- | --- |
| HOR / F_1_ | 0.651 | 0.098 | 0.012 |
| HOR / NOR | 0.580 | 0.091 | 0.002 |
| F_1_ / NOR | 0.890 | 0.155 | 0.782 |

**Supplemental Table 5** Number of adult offspring assigned to HOR, F_1_, and NOR parents, used to fit the offspring age at maturity model (**offspring** **n_AAM_**), and with body length measurements (**Offspring** **n_length_**). HOR are any hatchery-produced Chinook salmon collected at either the McKenzie or Leaburg Hatchery or Cougar Trap. F_1_s are first-generation, wild-born descendants of two HOR parents that were released above the dam in previous years. NOR are wild-born Chinook salmon that do not assign to a parent previously released above the dam and are presumed to be produced below the dam, on the mainstem, or elsewhere.

| **Parent Origin** | **Offspring n_AAM_** | **Offspring n_length_** |
| --- | --- | --- |
| HOR | 383 | 231 |
| F_1_ | 216 | 147 |
| NOR | 114 | 91 |

#

# Supplemental Figures


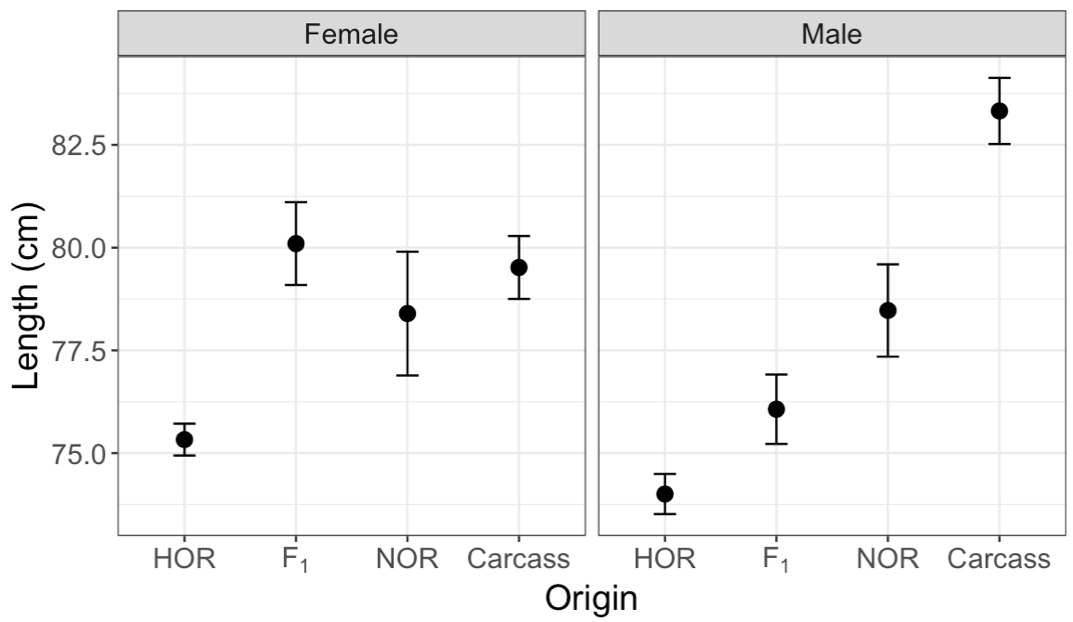


**Suppl. Fig. 1** Estimated marginal mean body length averaged over years for female (left panel) and male (right panel) HORs, F_1_s, NORs and Carcasses. HOR are any hatchery-produced Chinook salmon collected at either the McKenzie or Leaburg Hatchery or Cougar Trap. F_1_s are first-generation, wild-born descendants of two HOR parents that were released above the dam in previous years. NOR are wild-born Chinook salmon that do not assign to a parent previously released above the dam and are presumed to be produced below the dam, on the mainstem, or elsewhere. Carcass refers to a wild-born individual encountered during spawning ground surveys conducted on the South Fork McKenzie River below Cougar Dam or on the mainstem McKenzie River. Error bars are the 95% confidence interval of the estimated marginal mean.


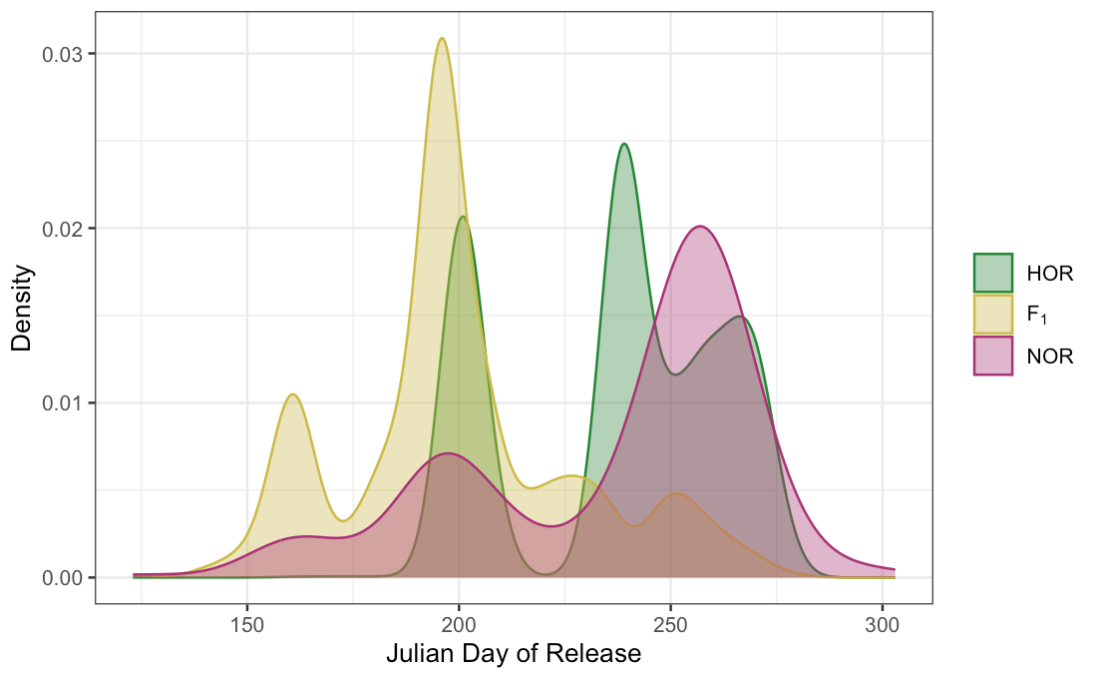


**Suppl. Fig. 2** Density plot of Julian Calendar day of release (*transport day*) for HORs, F_1_s, and NORs. HOR are any hatchery-produced Chinook salmon collected at either the McKenzie or Leaburg Hatchery or Cougar Trap. F_1_s are first-generation, wild-born descendants of two HOR parents that were released above the dam in previous years. NOR are wild-born Chinook salmon that do not assign to a parent previously released above the dam and are presumed to be produced below the dam, on the mainstem, or elsewhere.


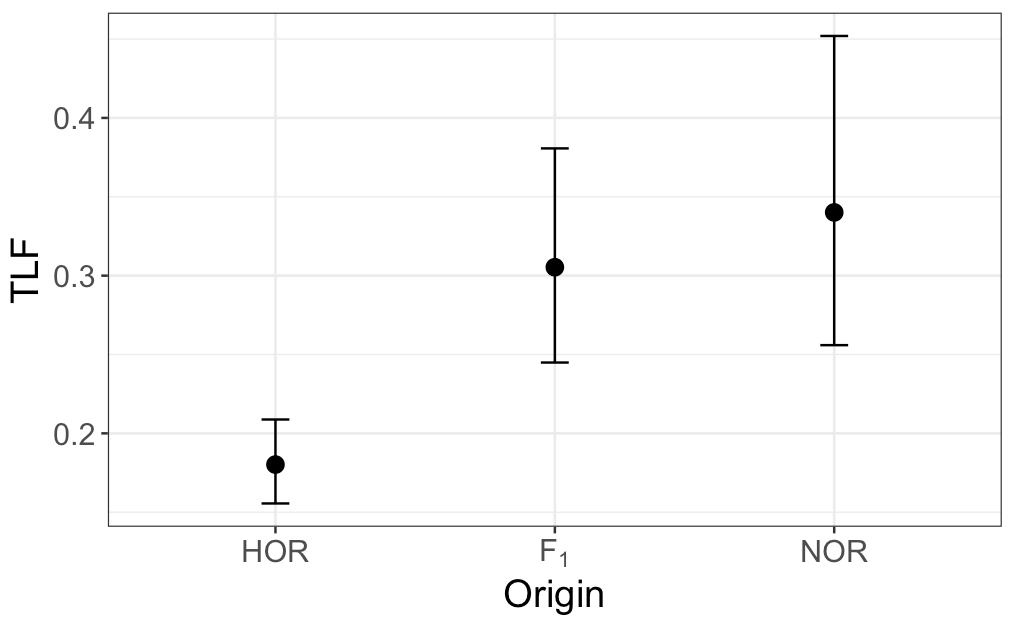


**Suppl. Fig. 3** Predicted effect of *origin* on total lifetime fitness (TLF) from the GLMM_TLF_ at typical levels of *length* and *year.* Error bars are standard errors of the predicted TLF. HOR are any hatchery-produced Chinook salmon collected at either the McKenzie or Leaburg Hatchery or Cougar Trap. F_1_s are first-generation, wild-born descendants of two HOR parents that were released above the dam in previous years. NOR are wild-born Chinook salmon that do not assign to a parent previously released above the dam and are presumed to be produced below the dam, on the mainstem, or elsewhere.

**
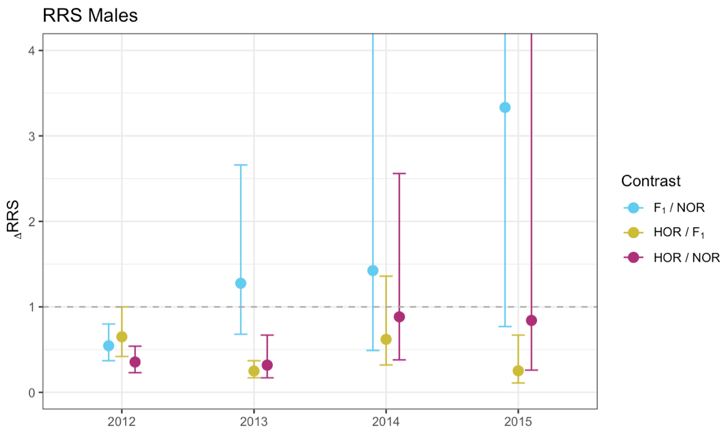
**

**
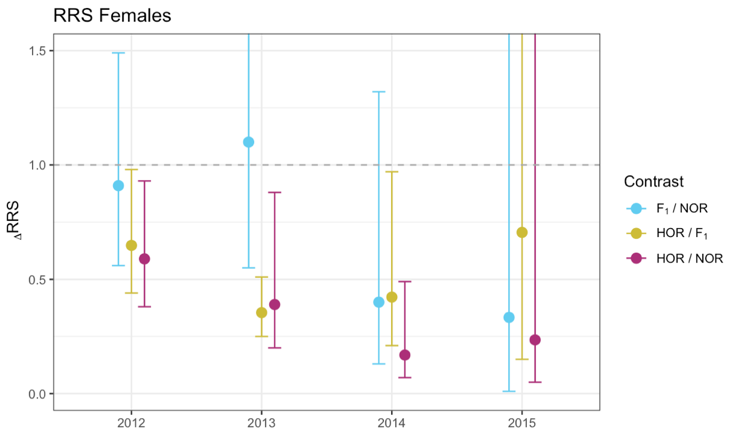
**

**Supplemental Fig. 4** _Δ_RRS by sex and year. Ratio between mean total lifetime fitness between different groups (_Δ_RRS). Male _Δ_RRS is in the top panel. Female _Δ_RRS is in the bottom panel. Mean TLF of HORs divided by mean TLF of either F_1_s or NORs. Error bars are maximum likelihood based 95% confidence intervals. Error bars extend beyond plot extent in some contrasts. HOR are any hatchery-produced Chinook salmon collected at either the McKenzie or Leaburg Hatchery or Cougar Trap. F_1_s are first-generation, wild-born descendants of two HOR parents that were released above the dam in previous years. NOR are wild-born Chinook salmon that do not assign to a parent previously released above the dam and are presumed to be produced below the dam, on the mainstem, or elsewhere.
