# Supplementary material for: A single generation in the wild increases fitness for descendants of hatchery‐origin Chinook salmon (Oncorhynchus tshawytscha)
Source: Evol Appl. 2024 Apr 11;17(4):e13678. doi: 10.1111/eva.13678 (PMC11009425; doi:10.1111/eva.13678)
Supplement: Supplementary file 2 — Appendix S2. [file EVA-17-e13678-s001.html]

Naturalization Analysis Notebook


Code 

- Show All Code
- Hide All Code

# Naturalization Analysis Notebook

```
require(car)
require(DHARMa)
require(emmeans)
require(MASS)
require(effects)
require(glmmTMB)
require(lme4)
require(kableExtra)
require(gt)
require(gtsummary)
require(tidyverse)
require(magrittr)
require(countreg)
require(lmerTest)
require(lubridate)
require(khroma)
```

# Summary

This notebook contains a log of all analyses associated with the manuscript titled: A single generation in the wild increases fitness for descendants of hatchery Chinook salmon (*Oncorhynchus tshawytscha*)

This is an R notebook. The .html version of this file is a fully rendered and interactive log. To view it, save the html and open in a browse. The .rmd version can be opened within R studio. To reproduce results or edit the analysis: clone the full repository onto your local machine and open the r project in rstudio. This will provide all needed data and objects.

# Pedigrees, Cohorts, Variables and Rationale of Comparisons

This notebook relies on a pedigree of all Chinook Salmon released above Cougar Dam on the South Fork McKenzie River from 2007 - 2017 using potential offspring sampled from 2010 to 2020.

## Summary of Cohort Years

Nearly all (98%) Chinook salmon on the South Fork McKenzie express an age at maturity of 4 - 5 years, with approximately 2% returning at age 3 or age 6. Therefore, our data data allow us to identify all offspring of salmon released above Cougar Dam in 2010 - 2014, and nearly all offspring of salmon released above Cougar Dam in 2015. From the offspring’s perspective, we sample all possible parents of potential offspring that return from 2013 - 2020, and nearly all (98%) parents of potential offspring that returned in 2012.

Therefore for adults that return and are released above the dam from 2012 - 2015, we know both their parentage (i.e. who are their parents) and how many returing adult offspring they produce.

## Definition of Variables

### TLF

For each candidate parent (any salmon released above the dam), we define total lifetime fitness (TLF) as the number of adult offspring produced by a individual candidate parent. Adult offspring are identified through parentage analysis, with any salmon collected at the Cougar Trap or during spawning ground surveys above or below the dam evaluated as potential offspring.

### Generation

This analysis is based on comparisons of TLF between three groups of candidate parent salmon released above Cougar Dam from 2012 - 2015:

**(1) Hatchery/HOR:** These are hatchery-origin fish from the McKenzie spring Chinook salmon hatchery program, that are released above the dam.  
**(2) F1:** We define F1s as the first generation wild born offspring of hatchery salmon released above the dam. Both of their parents are HOR.  
**(3) NORimmigrant:** We define NOR immigrant salmon as any salmon without an adipose clip that does not assign to a parent previously released above the dam. These salmon are presumed to be produced below the dam or elsewhere (no parentage assignment), and to also be wild born (no adipose clip, McKenzie clip rate > 99%). In the manuscript we will refer to these simply as NOR, but for the notebook we will label as NORimmigrant to avoid confusion with other unclipped fish.

We refer to this variable as **generation** in this notebook

**Other fish**  
There are many other individuals in the pedigree that do not fall into one of these three groups: \* Individuals that assign to a single parent  
\* Individuals from mixed mate pairs (e.g. HORxF1 parents)  
\* Individuals that are never released above the dam and therefore are not candidate parents and have no TLF estimate (e.g. F1 salmon that are recycled downstream of the dam and never return to be released above the dam)

To keep our conclusions as clear as possible we will ignore these other individuals and focus solely on HORs, F1s and NOR immigrants.

# Data

Here we import the finalized datasets from the McKenzie River Chinook salmon evaluation, create new datasets for this analysis, and summarize the data.

## Data Import

Here we import and assemble datasets from previous analyses

```
# load pedigree and full dataset
load("../input_data/full_filtered_dataset.R")
load("../input_data/pedigree.R")

# deduplicate (many individuals are sampled first live and later as carcasses, this defines individuals by their first encounter)
dedup <- full_data_1.0 %>%
  group_by(sample_id) %>%
  slice_max(date, with_ties = FALSE) %>%
  ungroup()
```

```
# let's get the metadata on the pedigree
pedigree_meta <- dedup %>%
  select(sample_id, year, type, date) %>%
  rename_with(.fn = ~ paste0("offspring_", .x)) %>%
  right_join(pedigree, by = c("offspring_sample_id" = "offspring_sample_id"))

#father
pedigree_meta <- dedup %>%
  select(sample_id, year, type, date) %>%
  rename_with(.fn = ~ paste0("father_", .x)) %>%
  right_join(pedigree_meta, by = c("father_sample_id" = "father")) %>%
  rename(father = father_sample_id)

#mother
pedigree_meta <- dedup %>%
  select(sample_id, year, type, date) %>%
  rename_with(.fn = ~ paste0("mother_", .x)) %>%
  right_join(pedigree_meta, by = c("mother_sample_id" = "mother")) %>%
  rename(mother = mother_sample_id)

pedigree_meta %<>%
  mutate(parent_year = (coalesce(father_year, mother_year)))

#let's add a column for the type of assignment, and one for combined types
pedigree_meta %<>%
  mutate(assn_type = case_when((mother == "none" & father == "none") ~ "none",
                               (mother == "none" & father != "none") ~ "male_only",
                               (mother != "none" & father == "none") ~ "female_only",
                               (mother != "none" & father != "none") ~ "pair",)) %>%
  mutate(parent_type = case_when((father_type == mother_type) ~ father_type,
                                 (is.na(father_type) & !(is.na(mother_type))) ~ mother_type,
                                  (is.na(mother_type) & !(is.na(father_type))) ~ father_type,
                                   (father_type != mother_type) ~ paste(mother_type, father_type, sep = "/")))
```

```
# let's not forget the "parents" dataset
parents <- dedup %>%
  filter(cand_parent == TRUE)

father_counts <- pedigree %>%
  group_by(father) %>%
  count() %>%
  rename(parent = father)

mother_counts <- pedigree %>%
  group_by(mother) %>%
  count() %>%
  rename(parent = mother)

parent_counts <- bind_rows(mother_counts, father_counts) 
rm(mother_counts)
rm(father_counts)

parents %<>%
  left_join(parent_counts, by = c("sample_id" = "parent")) %>%
  rename(tlf = n) %>%
  mutate(tlf = replace_na(tlf, 0))

rm(parent_counts)
```

```
# we will also rely heavily on the age at maturity dataset from the main project, let's make that R object here too
aam_data <- pedigree_meta %>%
  mutate(parent_year = (coalesce(father_year, mother_year))) %>%
  filter(!(is.na(parent_year))) %>%
  mutate(age = as.numeric(offspring_year) - as.numeric(parent_year)) %>%
  mutate(age = as.factor(age), offspring_year = as.factor(offspring_year))
```

```
# we also use the "mate pair" dataset, we will create this R object here as well

mate_pair <- pedigree_meta %>%
  filter(assn_type == "pair", parent_year < 2016, parent_year > 2009) %>%
  left_join(select(parents, sample_id, origin, tlf), by = c("father" = "sample_id")) %>%
  rename(father_origin = origin, father_tlf = tlf) %>%
  left_join(select(parents, sample_id, origin, tlf), by = c("mother" = "sample_id")) %>%
  rename(mother_origin = origin, mother_tlf = tlf) %>%
  mutate(cross = case_when(mother_origin == "NOR" & father_origin == "HOR" ~ "NxH",
                           mother_origin == "HOR" & father_origin == "NOR" ~ "HxN",
                           mother_origin == "NOR" & father_origin == "NOR" ~ "NxN",
                           mother_origin == "HOR" & father_origin == "HOR" ~ "HxH"))
mate_pair %<>%
  mutate(year_f = as.factor(parent_year),
         cross_f = as.factor(cross)) %>%
  mutate(cross_f = fct_relevel(cross_f, "NxN",   "HxN", "NxH","HxH"))
```

## Primary Dataset

Now let’s assemble and summarize the focal dataset for this project.

We must collect metadata and TLF, filter down to just our target individuals and assign the “generation” variable. We also know a priori that length, sex, and release date are all associated with TLF, so we will need to exclude any individuals where we do not know this information (n = 15, 12 of which are 2014 NOR immigrants).

```
pedigree_origin <- pedigree %>%
  left_join(select(dedup, sample_id, origin), by = c("mother" = "sample_id")) %>%
  rename(mother_origin = origin) %>%
  left_join(select(dedup, sample_id, origin), by = c("father" = "sample_id")) %>%
  rename(father_origin = origin)

F12_mmdata <- dedup %>%
  filter(year> 2011 , year < 2016, cand_parent == TRUE) %>%
  left_join(pedigree_origin, by = c("sample_id" = "offspring_sample_id")) %>%  # attach parents and their origin 
  mutate(generation = case_when(origin == "HOR" ~ "HOR",
                                mother_origin == "HOR" & father_origin == "HOR" ~"F1",
                                mother_origin == "NOR" & father_origin == "NOR" ~"F2", #see note 1 above for why this makes sense
                                mother == "none" & father == "none" ~ "NORimmigrant",
                                mother == "none" | father == "none" ~ "single_parentage",
                                mother_origin == "HOR" & father_origin == "NOR" ~"HORxNOR",
                                mother_origin == "NOR" & father_origin == "HOR" ~"NORxHOR")) %>%
  left_join(select(parents, sample_id, tlf) ) %>%
  mutate(jday = as.numeric(format(date, "%j"))) %>% #julian day in this case: days since the first day of the year
  mutate(jday_c = scale(jday, scale = F), #center the julian day to help with convergence
         sex = as.factor(sex),
         release= as.factor(release),
         year = as.factor(year),
         group = as.factor(paste(date, release, type))) %>%
  filter(length != 0, !(is.na(length))) %>%
  filter(generation %in% c("HOR", "F1", "NORimmigrant")) %>%
  mutate(generation  = fct_relevel(generation, c("HOR", "F1", "NORimmigrant")))
```

Great, now let’s summarise sample size.

```
kable(F12_mmdata %>%
  count(year, generation) %>%
  pivot_wider(id_cols = year, names_from = generation, values_from = n), caption = "Sample Size for the main dataset") %>% 
  kable_classic(full_width = F, html_font = "Arial")
```

Sample Size for the main dataset

| year | HOR | F1 | NORimmigrant |
| --- | --- | --- | --- |
| 2012 | 446 | 275 | 174 |
| 2013 | 454 | 127 | 26 |
| 2014 | 506 | 48 | 25 |
| 2015 | 619 | 15 | 12 |

Note that we do not have great balance between the years. Most F1s and NOR immigrants come from 2012. We know from the evaluation of the reintroduction that TLF varies between years, so we will need to be careful to investigate collinearity and other interactions between the year, generation and TLF variables.

# TLF Variation Model

Our principal question is whether there are fitness differences between HORs, F1s and NORimmigrants. To address this question we will fit a generalized linear model mixed on TLF. In addition to the effect of generation we will also explore covariates that we found were associated with TLF in the McKenzie River reintroduction. These include release length, sex, and julian day of release (fit as a linear continuous variable). We have some priors that suggest we should also explore some interactions: a sex \* generation interaction and a sex \* length interaction. We also fit year as fixed effect (too few to fit as random like we do in the evaluation: n\_year = 4). Finally we will include release group as a random effect. A release group is defined as set of individuals released together at the same location on the same day. Previous analyses have suggested that there is substantial variation in TLF among release groups, and the model fit benefits from the shrinkage associated with including release group.

## EDA

Even though we have already done this work for the full dataset, we will conduct a separate exploratory data analysis prior to fitting a model. This will include a careful examination of collinearity, identifying the correct distribution and link function for modeling, and model validation.

### Predictors

Let’s explore our predictor variables.

```
##################################################################
##################################################################
# this is a function to quickly produce biplots between our variables

panel.cor <- function(x, y, digits=1, prefix="", cex.cor = 6)
{
  usr <- par("usr"); on.exit(par(usr))
  par(usr = c(0, 1, 0, 1))
  r1=cor(x,y,use="pairwise.complete.obs")
  r <- abs(cor(x, y,use="pairwise.complete.obs"))
  txt <- format(c(r1, 0.123456789), digits=digits)[1]
  txt <- paste(prefix, txt, sep="")
  if(missing(cex.cor)) { cex <- 0.9/strwidth(txt) } else {
     cex = cex.cor}
  text(0.5, 0.5, txt, cex = cex * r)
}

##################################################################
panel.smooth2=function (x, y, col = par("col"), bg = NA, pch = par("pch"),
                        cex = 1, col.smooth = "black", span = 2/3, iter = 3, ...)
{
  points(x, y, pch = pch, col = col, bg = bg, cex = cex)
  ok <- is.finite(x) & is.finite(y)
  if (any(ok))
    lines(stats::lowess(x[ok], y[ok], f = span, iter = iter),
          col = 1, ...)
}

##################################################################
panel.lines2=function (x, y, col = par("col"), bg = NA, pch = par("pch"),
                       cex = 1, ...)
{
  points(x, y, pch = pch, col = col, bg = bg, cex = cex)
  ok <- is.finite(x) & is.finite(y)
  if (any(ok)){
    tmp=lm(y[ok]~x[ok])
    abline(tmp)}
}

##################################################################
panel.hist <- function(x, ...)
{
  usr <- par("usr"); on.exit(par(usr))
  par(usr = c(usr[1:2], 0, 1.5) )
  h <- hist(x, plot = FALSE)
  breaks <- h$breaks; nB <- length(breaks)
  y <- h$counts; y <- y/max(y)
  rect(breaks[-nB], 0, breaks[-1], y, col="white", ...)
}


F12_mmdata %>%
  select(tlf, sex, jday,  generation, year, length) %>%
  #mutate(tlf = log(tlf)) %>%
  pairs(., lower.panel = panel.cor, diag.panel = panel.hist, upper.panel = panel.smooth2)
```

In the figure above a histogram of each variable is along the diagonal. For the unlabeled factor vairables the levels are (in order) Sex(F, M), Generation (HOR, F1, NORimmigrant).

There are a handful of relationships that jump our even with a simple correlation. The most potentially problematic, as we already noted, is the relationship between generation and year. Generation is also weakly related to release day, sex and length. We will need to explore these in more detail than with this simple figure.

**Generation x Release Day**  
Let’s explore the relationship between release day and generation.

```
ggplot(data = F12_mmdata)+geom_density(aes(x = jday, fill = generation, color = generation), alpha = 0.4)+theme_bw()+scale_fill_manual(values = c("#228833", "#CCBB44", "#AA3377"))+scale_color_manual(values = c("#228833", "#CCBB44", "#AA3377"))+xlab("Julian Day of Release")
```

Yes, there is a relationship between generation and release day. Fortunately there is a lot of overlap so our model may be able to parse the effects and collinearity will not prevent us from making clear inferences.

**Generation x Sex**

```
ggplot(data = F12_mmdata)+geom_bar(aes(x = generation, fill = sex), position = "fill")+scale_fill_bright()+theme_bw()+ylab("Proportion")
```

HORs tend to be female biased relative to F1s and NORimmigrants. We will also need to be thoughtful about the relationship between these variables and TLF

**Generation x Length**

```
ggplot(data = F12_mmdata)+geom_density(aes(x = length, fill = generation, color = generation), alpha = 0.4)+theme_bw()+scale_fill_manual(values = c("#228833", "#CCBB44", "#AA3377"))+scale_color_manual(values = c("#228833", "#CCBB44", "#AA3377"))+xlab("Length (cm)")
```

```
anova(lm(length ~ generation + year, data = F12_mmdata))
```

```
plot(emmeans(lm(length ~ generation + year, data = F12_mmdata), "generation", type = "response"))
```

```
contrast(emmeans(lm(length ~ generation + year, data = F12_mmdata), "generation", type = "response"), "pairwise")
```

```
##  contrast           estimate    SE   df t.ratio p.value
##  HOR - F1             -2.997 0.367 2721  -8.175  <.0001
##  HOR - NORimmigrant   -3.715 0.484 2721  -7.679  <.0001
##  F1 - NORimmigrant    -0.718 0.535 2721  -1.342  0.3722
## 
## Results are averaged over the levels of: year 
## P value adjustment: tukey method for comparing a family of 3 estimates
```

HORs are significantly smaller than both F1s and NORimmigrants. F1s and NOR immigrants are the same length.

### Distribution

In all recent pedigree work on UWR reintroductions, a negative binomial distribution was a better fit to the data and/or more parsimonious fit than Poisson, QuasiPoisson, and various zero-inflation/hurdle models. However the zero-inflated negative binomial and negative binomial models were pretty close in performance. Let’s re-evaluate given that the zero-inflated model may perform better on this data subset.

For each of the two approaches (negbin and zero-inlfated negbin (zinb)) we’ll conduct model selection then we’ll compare the optimal models. Most of the model selection procedures I already have running won’t work well on the zero-inflated model so we’ll just follow the reccomendations of Zuur et al and rely on backwards stepwise Wald Tests, treating each component (zeros and conditional) separately. We will also ignore the random effects for now.

```
negbin <- glmmTMB(tlf ~ generation + length + sex+ jday_c+ year, data = F12_mmdata, family = nbinom2)
summary(negbin)
```

```
##  Family: nbinom2  ( log )
## Formula:          tlf ~ generation + length + sex + jday_c + year
## Data: F12_mmdata
## 
##      AIC      BIC   logLik deviance df.resid 
##   3275.5   3334.6  -1627.8   3255.5     2717 
## 
## 
## Dispersion parameter for nbinom2 family (): 0.733 
## 
## Conditional model:
##                         Estimate Std. Error z value Pr(>|z|)    
## (Intercept)            -6.919497   0.591420 -11.700  < 2e-16 ***
## generationF1            0.350368   0.142696   2.455 0.014075 *  
## generationNORimmigrant  0.592962   0.152888   3.878 0.000105 ***
## length                  0.067292   0.007337   9.171  < 2e-16 ***
## sexM                    0.054821   0.093884   0.584 0.559271    
## jday_c                 -0.003360   0.001750  -1.921 0.054776 .  
## year2013                0.700092   0.120731   5.799 6.68e-09 ***
## year2014               -0.025644   0.138051  -0.186 0.852637    
## year2015               -0.081733   0.154240  -0.530 0.596174    
## ---
## Signif. codes:  0 '***' 0.001 '**' 0.01 '*' 0.05 '.' 0.1 ' ' 1
```

```
# drop sex (p = 0.56, wald test)
negbin <- glmmTMB(tlf ~ generation + length + jday_c+ year , data = F12_mmdata, family = nbinom2)
summary(negbin)
```

```
##  Family: nbinom2  ( log )
## Formula:          tlf ~ generation + length + jday_c + year
## Data: F12_mmdata
## 
##      AIC      BIC   logLik deviance df.resid 
##   3273.8   3327.0  -1627.9   3255.8     2718 
## 
## 
## Dispersion parameter for nbinom2 family (): 0.732 
## 
## Conditional model:
##                         Estimate Std. Error z value Pr(>|z|)    
## (Intercept)            -6.874796   0.586633 -11.719  < 2e-16 ***
## generationF1            0.362240   0.141401   2.562   0.0104 *  
## generationNORimmigrant  0.607135   0.150995   4.021 5.80e-05 ***
## length                  0.067005   0.007324   9.149  < 2e-16 ***
## jday_c                 -0.003325   0.001750  -1.900   0.0574 .  
## year2013                0.697823   0.120719   5.781 7.44e-09 ***
## year2014               -0.027614   0.138022  -0.200   0.8414    
## year2015               -0.085282   0.154227  -0.553   0.5803    
## ---
## Signif. codes:  0 '***' 0.001 '**' 0.01 '*' 0.05 '.' 0.1 ' ' 1
```

```
# release day is marginal, let's keep it for now
```

```
zinb <- glmmTMB(tlf ~ jday_c + sex + generation +length +year , zi = ~ jday_c + sex + generation +length +year , data = F12_mmdata, family = nbinom2)
summary(zinb)
```

```
##  Family: nbinom2  ( log )
## Formula:          tlf ~ jday_c + sex + generation + length + year
## Zero inflation:       ~jday_c + sex + generation + length + year
## Data: F12_mmdata
## 
##      AIC      BIC   logLik deviance df.resid 
##   3260.7   3373.0  -1611.4   3222.7     2708 
## 
## 
## Dispersion parameter for nbinom2 family (): 0.956 
## 
## Conditional model:
##                         Estimate Std. Error z value Pr(>|z|)    
## (Intercept)            -6.791119   0.637190 -10.658  < 2e-16 ***
## jday_c                  0.001959   0.002184   0.897 0.369537    
## sexM                    0.052972   0.101544   0.522 0.601902    
## generationF1            0.419620   0.149538   2.806 0.005014 ** 
## generationNORimmigrant  0.522519   0.156951   3.329 0.000871 ***
## length                  0.066654   0.007801   8.544  < 2e-16 ***
## year2013                0.969310   0.138809   6.983 2.89e-12 ***
## year2014               -0.058377   0.147297  -0.396 0.691867    
## year2015               -0.127272   0.157069  -0.810 0.417771    
## ---
## Signif. codes:  0 '***' 0.001 '**' 0.01 '*' 0.05 '.' 0.1 ' ' 1
## 
## Zero-inflation model:
##                         Estimate Std. Error z value Pr(>|z|)   
## (Intercept)            -7.665119   5.431314  -1.411  0.15816   
## jday_c                  0.139582   0.054149   2.578  0.00994 **
## sexM                   -0.013771   0.603328  -0.023  0.98179   
## generationF1            3.818209   1.431247   2.668  0.00764 **
## generationNORimmigrant  0.234639   1.245773   0.188  0.85060   
## length                  0.003221   0.044768   0.072  0.94264   
## year2013                2.804217   1.530370   1.832  0.06690 . 
## year2014                0.891199   1.595979   0.558  0.57657   
## year2015               -8.192443  71.075076  -0.115  0.90824   
## ---
## Signif. codes:  0 '***' 0.001 '**' 0.01 '*' 0.05 '.' 0.1 ' ' 1
```

```
#sex and length have p-values ~ 1 in the zero part of the model, remove
zinb <- glmmTMB(tlf ~ jday_c + sex + generation +length +year , zi = ~ jday_c + generation  +year , data = F12_mmdata, family = nbinom2)
summary(zinb)
```

```
##  Family: nbinom2  ( log )
## Formula:          tlf ~ jday_c + sex + generation + length + year
## Zero inflation:       ~jday_c + generation + year
## Data: F12_mmdata
## 
##      AIC      BIC   logLik deviance df.resid 
##   3256.7   3357.2  -1611.4   3222.7     2710 
## 
## 
## Dispersion parameter for nbinom2 family (): 0.956 
## 
## Conditional model:
##                         Estimate Std. Error z value Pr(>|z|)    
## (Intercept)            -6.772952   0.588042 -11.518  < 2e-16 ***
## jday_c                  0.001982   0.002099   0.944 0.345063    
## sexM                    0.054178   0.093352   0.580 0.561666    
## generationF1            0.420176   0.147101   2.856 0.004285 ** 
## generationNORimmigrant  0.522552   0.156919   3.330 0.000868 ***
## length                  0.066432   0.007250   9.163  < 2e-16 ***
## year2013                0.968536   0.138544   6.991 2.73e-12 ***
## year2014               -0.059692   0.146460  -0.408 0.683590    
## year2015               -0.129179   0.154455  -0.836 0.402958    
## ---
## Signif. codes:  0 '***' 0.001 '**' 0.01 '*' 0.05 '.' 0.1 ' ' 1
## 
## Zero-inflation model:
##                         Estimate Std. Error z value Pr(>|z|)   
## (Intercept)             -7.36821    2.40246  -3.067  0.00216 **
## jday_c                   0.13934    0.04779   2.916  0.00355 **
## generationF1             3.80170    1.39880   2.718  0.00657 **
## generationNORimmigrant   0.22389    1.22836   0.182  0.85537   
## year2013                 2.75332    1.29097   2.133  0.03294 * 
## year2014                 0.84929    1.49261   0.569  0.56936   
## year2015               -12.92784  737.07027  -0.018  0.98601   
## ---
## Signif. codes:  0 '***' 0.001 '**' 0.01 '*' 0.05 '.' 0.1 ' ' 1
```

```
# next out is sex in the conditional
zinb <- glmmTMB(tlf ~ jday_c + generation +length +year , zi = ~ jday_c + generation  +year , data = F12_mmdata, family = nbinom2)
summary(zinb)
```

```
##  Family: nbinom2  ( log )
## Formula:          tlf ~ jday_c + generation + length + year
## Zero inflation:       ~jday_c + generation + year
## Data: F12_mmdata
## 
##      AIC      BIC   logLik deviance df.resid 
##   3255.1   3349.6  -1611.5   3223.1     2711 
## 
## 
## Dispersion parameter for nbinom2 family (): 0.955 
## 
## Conditional model:
##                         Estimate Std. Error z value Pr(>|z|)    
## (Intercept)            -6.730949   0.583900 -11.528  < 2e-16 ***
## jday_c                  0.002021   0.002104   0.961 0.336654    
## generationF1            0.431386   0.145932   2.956 0.003116 ** 
## generationNORimmigrant  0.536518   0.155205   3.457 0.000547 ***
## length                  0.066184   0.007241   9.140  < 2e-16 ***
## year2013                0.966005   0.138511   6.974 3.08e-12 ***
## year2014               -0.063487   0.146531  -0.433 0.664818    
## year2015               -0.133281   0.154645  -0.862 0.388769    
## ---
## Signif. codes:  0 '***' 0.001 '**' 0.01 '*' 0.05 '.' 0.1 ' ' 1
## 
## Zero-inflation model:
##                          Estimate Std. Error z value Pr(>|z|)   
## (Intercept)              -7.33423    2.40392  -3.051  0.00228 **
## jday_c                    0.13963    0.04801   2.908  0.00363 **
## generationF1              3.77084    1.39390   2.705  0.00683 **
## generationNORimmigrant    0.20510    1.22760   0.167  0.86731   
## year2013                  2.70982    1.28788   2.104  0.03537 * 
## year2014                  0.78777    1.49189   0.528  0.59747   
## year2015                -15.16859 2328.57532  -0.006  0.99480   
## ---
## Signif. codes:  0 '***' 0.001 '**' 0.01 '*' 0.05 '.' 0.1 ' ' 1
```

```
# now julian day in the conditional
zinb <- glmmTMB(tlf ~  generation +length +year , zi = ~ jday_c + generation  +year , data = F12_mmdata, family = nbinom2)
summary(zinb)
```

```
##  Family: nbinom2  ( log )
## Formula:          tlf ~ generation + length + year
## Zero inflation:       ~jday_c + generation + year
## Data: F12_mmdata
## 
##      AIC      BIC   logLik deviance df.resid 
##   3254.0   3342.6  -1612.0   3224.0     2712 
## 
## 
## Dispersion parameter for nbinom2 family (): 0.922 
## 
## Conditional model:
##                         Estimate Std. Error z value Pr(>|z|)    
## (Intercept)            -6.728544   0.583567 -11.530  < 2e-16 ***
## generationF1            0.361337   0.125961   2.869 0.004122 ** 
## generationNORimmigrant  0.536489   0.153815   3.488 0.000487 ***
## length                  0.066156   0.007245   9.131  < 2e-16 ***
## year2013                0.945585   0.136206   6.942 3.86e-12 ***
## year2014               -0.055193   0.144443  -0.382 0.702380    
## year2015               -0.143103   0.153281  -0.934 0.350512    
## ---
## Signif. codes:  0 '***' 0.001 '**' 0.01 '*' 0.05 '.' 0.1 ' ' 1
## 
## Zero-inflation model:
##                          Estimate Std. Error z value Pr(>|z|)   
## (Intercept)              -8.18147    2.58139  -3.169  0.00153 **
## jday_c                    0.14985    0.05019   2.986  0.00283 **
## generationF1              3.98235    1.54102   2.584  0.00976 **
## generationNORimmigrant    0.31799    1.31863   0.241  0.80944   
## year2013                  3.06659    1.49523   2.051  0.04027 * 
## year2014                  1.00409    1.73532   0.579  0.56285   
## year2015                -15.09148 2346.09962  -0.006  0.99487   
## ---
## Signif. codes:  0 '***' 0.001 '**' 0.01 '*' 0.05 '.' 0.1 ' ' 1
```

```
# this looks pretty good
```

Final ZINB model by backward stepwise selection using Wald Tests is pretty interesting. The conditional part of the model includes an effect of generation, length, and year, while the additional zeros include generation day and year. At face value this suggests that release day affects fitness through the likelihood of reproducing at all, whereas length only matters if you do manage to spawn. This is the basic expectation for how this should work if we assume the zero part of the model largely predicts propensity for pre-spawn mortality whereas the conditional part predicts TLF once you do spawn.

Now let’s compare the the ZINB and NegBin models.

First we’ll look at model validation

```
simulateResiduals(negbin, plot = TRUE)
```

```
## Object of Class DHARMa with simulated residuals based on 250 simulations with refit = FALSE . See ?DHARMa::simulateResiduals for help. 
##  
## Scaled residual values: 0.8833189 0.5585012 0.2131827 0.8928749 0.3074773 0.05568139 0.7841304 0.5851172 0.1772982 0.6566701 0.1105444 0.68838 0.8852255 0.6461571 0.6268861 0.7803337 0.06028185 0.03876886 0.2802067 0.520426 ...
```

```
simulateResiduals(zinb, plot = TRUE)
```

```
## Object of Class DHARMa with simulated residuals based on 250 simulations with refit = FALSE . See ?DHARMa::simulateResiduals for help. 
##  
## Scaled residual values: 0.8860861 0.3415169 0.8554841 0.9076655 0.5378584 0.7534525 0.07024913 0.1368748 0.3072816 0.1536555 0.5930816 0.5762024 0.8107234 0.05952554 0.02173524 0.3002994 0.3933638 0.2059115 0.5031039 0.7490026 ...
```

Both model fits look good using simulated residuals.

Now let’s compare using AIC, BIC and likelihood ratio tests (the negbin model is nested in the zinb model)

```
anova(negbin, zinb)
```

ZINB best by AIC (delta AIC ~ 20) and likelihood ratio test, but worse by BIC.

So the more complex ZINB model probably provides a better fit to the data. But what do we get for all this added model complexity? Let’s examine the differences in the model fit on the response scale (TLF) using a hanging rootogram.

```
# refit the final models with a different software to look at rootograms
negbin <- glm.nb(tlf ~  generation +length+ jday_c+year, data = F12_mmdata)

#rescale jday since this software has convergance issues
F12_mmdata %<>% mutate(jday_cs = scale(jday_c))
zinb <- zeroinfl(tlf ~  generation +length +year | jday_cs + generation +year , data = F12_mmdata, dist = "negbin")

rootogram(negbin, main = "Negative Binomial")
```

```
rootogram(zinb, main = "Zero-Inflated Negative Binomial")
```

These are nearly equivalent fits at all TLFs except 1. The negbin model is able to predict approximately the same number of zeros the ZINB model, without wildly differing at non-zeros. To me this suggests there isn’t a severe zero-inflation problem and the same processes might drive fitness at both zeros and non-zeros.

Let’s think about it this way, if both models offer similar explanatory power the benefit of the more complex model is additional inference between zero generating and non-zero generation processes (i.e. jday influences PSM whereas length influences TLF once you successfully survive to spawning time). Are we so confident in our model selection procedure that we should stake an entire conclusion on it? How much better is a model with exactly the same variables on both sides of the conditional/zero portions with respect to information/likelihood(AIC, LRT)

```
zinb <- glmmTMB(tlf ~  generation +length +year , zi = ~ jday_c + generation  +year , data = F12_mmdata, family = nbinom2)

# here fit a model where the zero and conditional effects are the same and compare 
zinb2 <- glmmTMB(tlf ~ jday_c+ generation +length +year, zi = ~ jday_c + generation +length +year  , data = F12_mmdata, family = nbinom2)

# and compare
AIC(zinb, zinb2)
```

```
anova(zinb, zinb2)
```

Delta AIC is ~4, fails to be different in likelihood ratio test. This suggests the more complex model isn’t worth it. Any decent results section would beed to consider both models above (with and without differences between zero and conditional sections).  
Instead we should defer to the negbin model. There is nothing wrong with model fit, it is nearly as good of a fit as the zinb, and it is much easier to get our central message across without having to explain the added complexity of model selection in a hurdle/zero-inflation model.

### Collinearity

Great, we know we want to use a negative binomial, and we know which variables might be collinear. Let’s see if there is enough information to parse the effects of year, generation and sex on TLF. If we find strong collinearity then our power will be low, our estimates will have large SEs and the inferences we can draw will be limited.

We will do this using the VIF (or more specifically GVIF^(1/2\*df)).

```
# fit beyond optimal model (all possible fixed effect)
beyond_opt <- glm.nb(tlf ~ generation + length + sex+ jday_c+ year , data = F12_mmdata)
vif(beyond_opt)
```

```
##                GVIF Df GVIF^(1/(2*Df))
## generation 1.988781  2        1.187536
## length     1.215163  1        1.102344
## sex        1.059009  1        1.029082
## jday_c     1.512096  1        1.229673
## year       1.419041  3        1.060065
```

Very low GVIFs! This is a relief considering the relationship between release day/generation, length/generation and year/generation. Despite these relationships, there is limited correlation between the estimates of their effects and we are likely to have sufficient power/information to identify their independent relationships with TLF.

We are clear to move on to model selection.

## Model Selection and Validation

Let’s conduct model selection.

First we will examine the random effect structure, using REML. There is only a single random effect (release group). So, here we compare a mixed model with release group to it’s equivalent glm with no random effects.

```
beyond_opt_mm <- glmmTMB(tlf ~ generation + length + sex+ jday_c+ year + (1| group ) , data = F12_mmdata, family = nbinom2, REML = TRUE) 
beyond_opt <- glmmTMB(tlf ~ generation + length + sex+ jday_c+ year  , data = F12_mmdata, family = nbinom2, REML = TRUE)

AIC(beyond_opt_mm, beyond_opt)
```

```
BIC(beyond_opt_mm, beyond_opt)
```

Mixed model is a better performer. We will now switch to model selection for the fixed effect structure and fit using ML. The method is backward stepwise selection using likelihood ratio tests, and a cutoff of p = 0.05.

```
beyond_opt_mm <- glmmTMB(tlf ~ generation*sex + length+ sex* jday_c+ year + (1| group ) , data = F12_mmdata, family = nbinom2) 
drop1(beyond_opt_mm, test = "Chisq")
```

```
#drop sex*release day interaction

beyond_opt_mm <- glmmTMB(tlf ~ generation*sex + length + sex+ jday_c+ year + (1| group ) , data = F12_mmdata, family = nbinom2) 
drop1(beyond_opt_mm, test = "Chisq")
```

```
#drop generation*sex interaction

beyond_opt_mm <- glmmTMB(tlf ~ generation + length + sex+ jday_c+ year + (1| group ) , data = F12_mmdata, family = nbinom2) 
drop1(beyond_opt_mm, test = "Chisq")
```

```
#drop sex (p value = 0.63)
mm_f12 <- glmmTMB(tlf ~ generation + length +  jday_c+ year + (1| group ) , data = F12_mmdata, family = nbinom2) 
drop1(mm_f12, test = "Chisq")
```

```
# Day of release only marginally improves model fit (delta AIC = 1.8, LRT p value = 0.053). Let's drop it.
mm_f12 <- glmmTMB(tlf ~ generation + length +   year + (1| group ) , data = F12_mmdata, family = nbinom2) 
drop1(mm_f12, test = "Chisq")
```

We dropped (in order) sex \* jday, sex \* generation, sex, and day of release. Now let’s check the fit using simulated residuals.

```
simulateResiduals(mm_f12, plot = TRUE)
```

```
## Object of Class DHARMa with simulated residuals based on 250 simulations with refit = FALSE . See ?DHARMa::simulateResiduals for help. 
##  
## Scaled residual values: 0.03246341 0.4773073 0.1690777 0.7591336 0.1899933 0.3279656 0.7618147 0.4880879 0.1689211 0.5639646 0.07723448 0.6276607 0.9737457 0.679942 0.531472 0.411658 0.8752665 0.815412 0.1754228 0.1984262 ...
```

Model fit is excellent.

## Final Model

```
summary(mm_f12)
```

```
##  Family: nbinom2  ( log )
## Formula:          tlf ~ generation + length + year + (1 | group)
## Data: F12_mmdata
## 
##      AIC      BIC   logLik deviance df.resid 
##   3273.1   3326.3  -1627.5   3255.1     2718 
## 
## Random effects:
## 
## Conditional model:
##  Groups Name        Variance Std.Dev.
##  group  (Intercept) 0.03982  0.1995  
## Number of obs: 2727, groups:  group, 132
## 
## Dispersion parameter for nbinom2 family (): 0.763 
## 
## Conditional model:
##                         Estimate Std. Error z value Pr(>|z|)    
## (Intercept)            -6.941631   0.590009 -11.765  < 2e-16 ***
## generationF1            0.527212   0.133406   3.952 7.75e-05 ***
## generationNORimmigrant  0.635016   0.163067   3.894 9.85e-05 ***
## length                  0.067076   0.007339   9.140  < 2e-16 ***
## year2013                0.707712   0.141223   5.011 5.41e-07 ***
## year2014               -0.027883   0.160516  -0.174    0.862    
## year2015               -0.032724   0.183914  -0.178    0.859    
## ---
## Signif. codes:  0 '***' 0.001 '**' 0.01 '*' 0.05 '.' 0.1 ' ' 1
```

Generation (F0, F1, F2 and NORimmigrant), year (fixed effect factor four years) and length significantly improve the fit to the data according to LRT, AIC and Wald tests.

Let’s look at the predicted effects

```
eff1 <- predictorEffect("generation", mm_f12)
effdf <- as.data.frame(eff1)
effdf$generation <- factor(effdf$generation, levels=c("HOR", "F1", "NORimmigrant")) # relevel the genertions for a nicer plot

#note that this throws an error. w/r/t this error the glmmTMB author (Ben Bolker) states that "the predicted variances are used when computing residuals (which are Pearson residuals by default) for partial residuals plots. I think that if you're not plotting partial residuals, it doesn't matter." 
# Since we do not plot partial residuals but instead th95 CI for the predition, we are good here

ggplot(data = effdf, aes(x = (generation), y = fit))+ 
  geom_point(position=position_dodge(width=0.3), size = 3) + 
  geom_errorbar(aes(ymin = lower, ymax = upper), position=position_dodge(width=0.3), width = 0.1)+ylab("TLF")+xlab("Generation")+theme_bw()
```

Let’s do some post hoc testing analysis using marginal means in the emmeans packge.

We should think a lot and be very clear about how to weight observations across different levels of the other predictors here given the lack of balance in the data. For example, “classic” estimated marginal means where each level of year are given equal weights do not seem appropriate. We do not have an experiment so much as an observational dataset: variation in sample sizes between years and generation are not reflective of some kind of sampling process, but the biological reality in the river. Therefore we should not give equal weighting to the marginal mean for NOR fitness in 2015 where sample size is just 7% of what it is in 2012. We will use the “cell” based weighting scheme of the emmeans “reference grid,” but we should also see how sensitive our results are to this decision and also use the default where everything is weighted equally (e.g. estimated marginal means)

```
# we'll use emmeans for this
em <- emmeans(mm_f12, "generation", weights = "cell")
contrast(em, "pairwise", adjust = "Tukey", type = "response")
```

```
##  contrast           ratio     SE   df null t.ratio p.value
##  HOR / F1           0.563 0.0732 2718    1  -4.417  <.0001
##  HOR / NORimmigrant 0.568 0.0888 2718    1  -3.621  0.0009
##  F1 / NORimmigrant  1.009 0.1625 2718    1   0.053  0.9985
## 
## Results are averaged over the levels of: year 
## P value adjustment: tukey method for comparing a family of 3 estimates 
## Tests are performed on the log scale
```

The novel finding here is that both NORimmigrants and F1s have greater fitness than HORs, but do not differ from one another.

# TLF Variation Kalinowski CIs

Our primary hypotheis testing method is to fit generalized linear mixed model that includes the factor “generation” as a fixed effect and then determine if the estimated effect of the different levels of this factor are significantly different from one another, once all other effects have been controlled for. This is likely the best way to address our questions given that the covariates length and year have such large effects on fitness, but let’s not forget the clever approach that has been employed in past studies of RRS.

The delta method takes advantage of the fact while TLF is a count variable with variance greater than the mean and requiring very advanced statistics to appropriately model, RRS is a ratio. If we are able to describe mean and variance of TLF for each of the groups we should be able to divide them by one another and generate a ratio, and a confidence interval for this ratio can be estimated using the a bit of clever math and likelihoods. If the confidence interval doesn’t include one, then the fitness of the groups is different!

Let’s use this approach for each year.

## Overall RRS

```
# kalinowski method
# rather than re-invent the wheel here let's make sure my interpretation of the Kalinowski paper is correct and modify seom existing code from a reviewed paper. My understanding matched the modified code here, so I just used that. We should be sure to cite if it winds up in a paper.
# https://doi.org/10.1098/rsos.221271

rrs_ci_kalinowski_auke <- function(n_h_off, n_w_off, n_h_par, n_w_par, alpha){
  chi_alpha <- qchisq(p = (1 - alpha), df = 1)
  n_off <- sum(c(n_h_off, n_w_off))
  n_par <- sum(c(n_h_par, n_w_par))
  
  rs_h <- n_h_off / n_h_par
  rs_w <- n_w_off / n_w_par
  
  p_h_par <- n_h_par / n_par
  p_w_par <- n_w_par / n_par
  
  rrs_h <- rs_h / rs_w
  rrs_w <- rs_w / rs_w
  rrs_avg <- (rrs_h * p_h_par) + (rrs_w * p_w_par)
  
  rrs_ml <- (n_h_off * log(p_h_par * rrs_h / rrs_avg)) + (n_w_off * log(p_w_par * rrs_w / rrs_avg))
  
  xi_dist <- bind_rows(
    lapply(seq(0.01, 50, by = 0.01), function(rrs_h_xi) { #upper RRS up to 50
      rrs_avg_xi <- (rrs_h_xi * p_h_par) + (rrs_w * p_w_par)
      tibble(rrs_crit = rrs_h_xi,
             logl = (n_h_off * log(p_h_par * rrs_h_xi / rrs_avg_xi)) + (n_w_off * log(p_w_par * rrs_w / rrs_avg_xi)) - (rrs_ml - chi_alpha / 2)
      )
    } )
  )
  
  rrs_min <- xi_dist %>% 
    mutate(abs_logl = abs(logl)) %>% 
    filter(rrs_crit < rrs_h) %>% 
    top_n(-1, abs_logl) %>% 
    pull(rrs_crit)
  
  rrs_max <- xi_dist %>% 
    mutate(abs_logl = abs(logl)) %>% 
    filter(rrs_crit > rrs_h) %>% 
    top_n(-1, abs_logl) %>% 
    pull(rrs_crit)
  
  return(c(rrs_min, rrs_h, rrs_max))
}

# get data to enter
rrs_delta <- F12_mmdata %>%
  group_by(generation, year) %>%
  summarise(n = n(), n_offspring = sum(tlf)) %>%
  pivot_wider(names_from = generation, values_from = c(n, n_offspring))


#n_h_off, n_w_off, n_h_par, n_w_par, alpha


# embarrisingly redundant, slow code, consider revising, can be made at least 3x faster by not calling the same function 3 times witht e same parameters...
# argument order reminder of function, n_h_off, n_w_off, n_h_par, n_w_par, alpha
rrs_delta %<>%
  rowwise() %>%
  mutate(rrs_NH_lwr = rrs_ci_kalinowski_auke(n_offspring_HOR, n_offspring_NORimmigrant, n_HOR, n_NORimmigrant, alpha = 0.05)[1],
         rrs_NH = rrs_ci_kalinowski_auke(n_offspring_HOR, n_offspring_NORimmigrant, n_HOR, n_NORimmigrant, alpha = 0.05)[2],
         rrs_NH_upr = rrs_ci_kalinowski_auke(n_offspring_HOR, n_offspring_NORimmigrant, n_HOR, n_NORimmigrant, alpha = 0.05)[3]) %>%
  mutate(rrs_FH_lwr = rrs_ci_kalinowski_auke(n_offspring_HOR, n_offspring_F1, n_HOR, n_F1, alpha = 0.05)[1],
         rrs_FH = rrs_ci_kalinowski_auke(n_offspring_HOR, n_offspring_F1, n_HOR, n_F1, alpha = 0.05)[2],
         rrs_FH_upr = rrs_ci_kalinowski_auke(n_offspring_HOR, n_offspring_F1, n_HOR, n_F1, alpha = 0.05)[3]) %>%
  mutate(rrs_FN_lwr = rrs_ci_kalinowski_auke(n_offspring_F1, n_offspring_NORimmigrant, n_F1, n_NORimmigrant, alpha = 0.05)[1],
         rrs_FN = rrs_ci_kalinowski_auke(n_offspring_F1, n_offspring_NORimmigrant, n_F1, n_NORimmigrant, alpha = 0.05)[2],
         rrs_FN_upr = rrs_ci_kalinowski_auke(n_offspring_F1, n_offspring_NORimmigrant, n_F1, n_NORimmigrant, alpha = 0.05)[3])
```

Now let’s plot these.

```
plot_data <- rrs_delta %>%
  select(year, starts_with("rrs")) %>%
  pivot_longer(cols = -c(year), names_prefix = "rrs_", names_to = c("numerator", "value_type"), names_sep = "_", values_to = "value") %>%
  mutate(value_type = case_when(is.na(value_type) ~ "MeanRRS",
                                TRUE ~ value_type)) %>%
  pivot_wider(names_from = "value_type", values_from = "value") %>%
  mutate(numerator = as.factor(numerator),
         numerator = fct_relevel(numerator, "FN", "FH", "NH")) 

ggplot(plot_data)+geom_errorbar(aes(x = year, y = MeanRRS, ymin = lwr, ymax = upr, color = numerator), width = 0.3, position = position_dodge())+ geom_hline(aes(yintercept = 1), linetype = 2, color = "darkgrey")+theme_bw()+geom_point(aes(x = year, y = MeanRRS, color = numerator), size = 3,  position = position_dodge(width = 0.3))+xlab("")+scale_color_manual(name = "Contrast", values = c("#66CCEE", "#CCBB44", "#AA3377"), labels = c(expression(F[1]*" / NOR"),expression("HOR / " *F[1]), "HOR / NOR" ))+ylab(expression(" "[Delta]*"RRS"))+ theme(legend.text.align = 0)
```

```
ggplot(plot_data)+geom_errorbar(aes(x = year, y = MeanRRS, ymin = lwr, ymax = upr, color = numerator), width = 0.3, position = position_dodge())+ geom_hline(aes(yintercept = 1), linetype = 2, color = "darkgrey")+theme_bw()+geom_point(aes(x = year, y = MeanRRS, color = numerator), size = 3,  position = position_dodge(width = 0.3))+xlab("")+scale_color_manual(name = "Contrast", values = c("#66CCEE", "#CCBB44", "#AA3377"), labels = c(expression(F[1]*" / NOR"),expression("HOR / " *F[1]), "HOR / NOR" ))+ylab(expression(" "[Delta]*"RRS"))+ theme(legend.text.align = 0) + coord_cartesian(ylim=c(0, 2))
```

```
#ggplot(plot_data)+geom_pointrange(aes(x = year, y = MeanRRS, ymin = lwr, ymax = upr, color = numerator), width = 0.3, position = position_dodge())+ geom_hline(aes(yintercept = 1), linetype = 2, color = "red")+theme_bw()

#ggplot(plot_data)+geom_errorbar(aes(x = year, y = MeanRRS, ymin = lwr, ymax = upr, color = numerator), width = 0.3, position = position_dodge(width = 0.5))+ geom_hline(aes(yintercept = 1), linetype = 2, color = "darkgrey")+theme_bw()+geom_point(aes(x = year, y = MeanRRS, color = numerator), size = 4,  position = position_dodge(width = 0.5))+xlab("")+scale_color_manual(name = "Contrast", values = c("#66CCEE", "#CCBB44", "#AA3377"), labels = c(expression(F[1]*" / NOR"),expression("HOR / " *F[1]), "HOR / NOR" ))+ylab(expression(" "[Delta]*"RRS"))+ theme(legend.text.align = 0) + coord_cartesian(ylim=c(0, 2))+theme(axis.text = element_text(size = 14), axis.title = element_text(size = 16), legend.text = element_text(size = 14), legend.title = element_text(size = 14))
```

For the HOR RRSs, same results within each year (except 2015) using the delta method. RRS < 1 for regardless of whether F1s or NOR immigrants are in the numerator. Note that that this RRS calculation can’t take differences in covariates like length and release day into effect.

Also note one important difference F1/NOR fitness is less than one in 2012.

```
#Let's also calculate an overall RRS. Note that because some years have higher fitness than others, the unbalanced sample sizes across generation and year will create some major biases here and we shouldn't present this. 

rrs_delta_all <- F12_mmdata %>%
  group_by(generation) %>%
  summarise(n = n(), n_offspring = sum(tlf)) %>%
  pivot_wider(names_from = generation, values_from = c(n, n_offspring))

rrs_delta_all %<>%
  rowwise() %>%
  mutate(rrs_NH_lwr = rrs_ci_kalinowski_auke(n_offspring_HOR, n_offspring_NORimmigrant, n_HOR, n_NORimmigrant, alpha = 0.05)[1],
         rrs_NH = rrs_ci_kalinowski_auke(n_offspring_HOR, n_offspring_NORimmigrant, n_HOR, n_NORimmigrant, alpha = 0.05)[2],
         rrs_NH_upr = rrs_ci_kalinowski_auke(n_offspring_HOR, n_offspring_NORimmigrant, n_HOR, n_NORimmigrant, alpha = 0.05)[3]) %>%
  mutate(rrs_FH_lwr = rrs_ci_kalinowski_auke(n_offspring_HOR, n_offspring_F1, n_HOR, n_F1, alpha = 0.05)[1],
         rrs_FH = rrs_ci_kalinowski_auke(n_offspring_HOR, n_offspring_F1, n_HOR, n_F1, alpha = 0.05)[2],
         rrs_FH_upr = rrs_ci_kalinowski_auke(n_offspring_HOR, n_offspring_F1, n_HOR, n_F1, alpha = 0.05)[3]) %>%
  mutate(rrs_FN_lwr = rrs_ci_kalinowski_auke(n_offspring_F1, n_offspring_NORimmigrant, n_F1, n_NORimmigrant, alpha = 0.05)[1],
         rrs_FN = rrs_ci_kalinowski_auke(n_offspring_F1, n_offspring_NORimmigrant, n_F1, n_NORimmigrant, alpha = 0.05)[2],
         rrs_FN_upr = rrs_ci_kalinowski_auke(n_offspring_F1, n_offspring_NORimmigrant, n_F1, n_NORimmigrant, alpha = 0.05)[3])
```

## Sex Specific RRS

We also chose not to include sex specific RRS because sex and sex\*generation interaction were not retained in the final model, but let’s also split the delta method results up by sex and plot again to keep with conventions.

**Males**

```
# get data to enter
rrs_delta_males <- F12_mmdata %>%
  filter(sex == "M") %>%
  group_by(generation, year) %>%
  summarise(n = n(), n_offspring = sum(tlf)) %>%
  pivot_wider(names_from = generation, values_from = c(n, n_offspring))
```

```
## `summarise()` has grouped output by 'generation'. You can override using the
## `.groups` argument.
```

```
#n_h_off, n_w_off, n_h_par, n_w_par, alpha


# embarrisingly redundant, slow code, consider revising, can be made at least 3x faster by not calling the same function 3 times witht e same parameters...
# argument order reminder of function, n_h_off, n_w_off, n_h_par, n_w_par, alpha
rrs_delta_males %<>%
  rowwise() %>%
  mutate(rrs_NH_lwr = rrs_ci_kalinowski_auke(n_offspring_HOR, n_offspring_NORimmigrant, n_HOR, n_NORimmigrant, alpha = 0.05)[1],
         rrs_NH = rrs_ci_kalinowski_auke(n_offspring_HOR, n_offspring_NORimmigrant, n_HOR, n_NORimmigrant, alpha = 0.05)[2],
         rrs_NH_upr = rrs_ci_kalinowski_auke(n_offspring_HOR, n_offspring_NORimmigrant, n_HOR, n_NORimmigrant, alpha = 0.05)[3]) %>%
  mutate(rrs_FH_lwr = rrs_ci_kalinowski_auke(n_offspring_HOR, n_offspring_F1, n_HOR, n_F1, alpha = 0.05)[1],
         rrs_FH = rrs_ci_kalinowski_auke(n_offspring_HOR, n_offspring_F1, n_HOR, n_F1, alpha = 0.05)[2],
         rrs_FH_upr = rrs_ci_kalinowski_auke(n_offspring_HOR, n_offspring_F1, n_HOR, n_F1, alpha = 0.05)[3]) %>%
  mutate(rrs_FN_lwr = rrs_ci_kalinowski_auke(n_offspring_F1, n_offspring_NORimmigrant, n_F1, n_NORimmigrant, alpha = 0.05)[1],
         rrs_FN = rrs_ci_kalinowski_auke(n_offspring_F1, n_offspring_NORimmigrant, n_F1, n_NORimmigrant, alpha = 0.05)[2],
         rrs_FN_upr = rrs_ci_kalinowski_auke(n_offspring_F1, n_offspring_NORimmigrant, n_F1, n_NORimmigrant, alpha = 0.05)[3])
```

```
plot_data <- rrs_delta_males %>%
  select(year, starts_with("rrs")) %>%
  pivot_longer(cols = -c(year), names_prefix = "rrs_", names_to = c("numerator", "value_type"), names_sep = "_", values_to = "value") %>%
  mutate(value_type = case_when(is.na(value_type) ~ "MeanRRS",
                                TRUE ~ value_type)) %>%
  pivot_wider(names_from = "value_type", values_from = "value") %>%
  mutate(numerator = as.factor(numerator),
         numerator = fct_relevel(numerator, "FN", "FH", "NH")) 
  
ggplot(plot_data)+geom_errorbar(aes(x = year, y = MeanRRS, ymin = lwr, ymax = upr, color = numerator), width = 0.3, position = position_dodge())+ geom_hline(aes(yintercept = 1), linetype = 2, color = "darkgrey")+theme_bw()+geom_point(aes(x = year, y = MeanRRS, color = numerator), size = 3,  position = position_dodge(width = 0.3))+xlab("")+scale_color_manual(name = "Contrast", values = c("#66CCEE", "#CCBB44", "#AA3377"), labels = c(expression(F[1]*" / NOR"),expression("HOR / " *F[1]), "HOR / NOR" ))+ylab(expression(" "[Delta]*"RRS"))+ theme(legend.text.align = 0) +ggtitle("RRS Males")
```

```
#ggplot(plot_data)+geom_pointrange(aes(x = year, y = MeanRRS, ymin = lwr, ymax = upr, color = numerator), width = 0.3, position = position_dodge())+ geom_hline(aes(yintercept = 1), linetype = 2, col

#kinda difficult to see 2012 CI let's zoom in 
ggplot(plot_data)+geom_errorbar(aes(x = year, y = MeanRRS, ymin = lwr, ymax = upr, color = numerator), width = 0.3, position = position_dodge())+ geom_hline(aes(yintercept = 1), linetype = 2, color = "darkgrey")+theme_bw()+geom_point(aes(x = year, y = MeanRRS, color = numerator), size = 3,  position = position_dodge(width = 0.3))+xlab("")+scale_color_manual(name = "Contrast", values = c("#66CCEE", "#CCBB44", "#AA3377"), labels = c(expression(F[1]*" / NOR"),expression("HOR / " *F[1]), "HOR / NOR" ))+ylab(expression(" "[Delta]*"RRS"))+ theme(legend.text.align = 0) +ggtitle("RRS Males")+coord_cartesian(ylim=c(0,4))
```

**Females**

```
# get data to enter
rrs_delta_females <- F12_mmdata %>%
  filter(sex == "F") %>%
  group_by(generation, year) %>%
  summarise(n = n(), n_offspring = sum(tlf)) %>%
  pivot_wider(names_from = generation, values_from = c(n, n_offspring))
```

```
## `summarise()` has grouped output by 'generation'. You can override using the
## `.groups` argument.
```

```
#n_h_off, n_w_off, n_h_par, n_w_par, alpha


# embarrisingly redundant, slow code, consider revising, can be made at least 3x faster by not calling the same function 3 times witht e same parameters...
# argument order reminder of function, n_h_off, n_w_off, n_h_par, n_w_par, alpha
rrs_delta_females %<>%
  rowwise() %>%
  mutate(rrs_NH_lwr = rrs_ci_kalinowski_auke(n_offspring_HOR, n_offspring_NORimmigrant, n_HOR, n_NORimmigrant, alpha = 0.05)[1],
         rrs_NH = rrs_ci_kalinowski_auke(n_offspring_HOR, n_offspring_NORimmigrant, n_HOR, n_NORimmigrant, alpha = 0.05)[2],
         rrs_NH_upr = rrs_ci_kalinowski_auke(n_offspring_HOR, n_offspring_NORimmigrant, n_HOR, n_NORimmigrant, alpha = 0.05)[3]) %>%
  mutate(rrs_FH_lwr = rrs_ci_kalinowski_auke(n_offspring_HOR, n_offspring_F1, n_HOR, n_F1, alpha = 0.05)[1],
         rrs_FH = rrs_ci_kalinowski_auke(n_offspring_HOR, n_offspring_F1, n_HOR, n_F1, alpha = 0.05)[2],
         rrs_FH_upr = rrs_ci_kalinowski_auke(n_offspring_HOR, n_offspring_F1, n_HOR, n_F1, alpha = 0.05)[3]) %>%
  mutate(rrs_FN_lwr = rrs_ci_kalinowski_auke(n_offspring_F1, n_offspring_NORimmigrant, n_F1, n_NORimmigrant, alpha = 0.05)[1],
         rrs_FN = rrs_ci_kalinowski_auke(n_offspring_F1, n_offspring_NORimmigrant, n_F1, n_NORimmigrant, alpha = 0.05)[2],
         rrs_FN_upr = rrs_ci_kalinowski_auke(n_offspring_F1, n_offspring_NORimmigrant, n_F1, n_NORimmigrant, alpha = 0.05)[3])
```

```
plot_data <- rrs_delta_females %>%
  select(year, starts_with("rrs")) %>%
  pivot_longer(cols = -c(year), names_prefix = "rrs_", names_to = c("numerator", "value_type"), names_sep = "_", values_to = "value") %>%
  mutate(value_type = case_when(is.na(value_type) ~ "MeanRRS",
                                TRUE ~ value_type)) %>%
  pivot_wider(names_from = "value_type", values_from = "value") %>%
  mutate(numerator = as.factor(numerator),
         numerator = fct_relevel(numerator, "FN", "FH", "NH")) 
  
ggplot(plot_data)+geom_errorbar(aes(x = year, y = MeanRRS, ymin = lwr, ymax = upr, color = numerator), width = 0.3, position = position_dodge())+ geom_hline(aes(yintercept = 1), linetype = 2, color = "darkgrey")+theme_bw()+geom_point(aes(x = year, y = MeanRRS, color = numerator), size = 3,  position = position_dodge(width = 0.3))+xlab("")+scale_color_manual(name = "Contrast", values = c("#66CCEE", "#CCBB44", "#AA3377"), labels = c(expression(F[1]*" / NOR"),expression("HOR / " *F[1]), "HOR / NOR" ))+ylab(expression(" "[Delta]*"RRS"))+ theme(legend.text.align = 0) +ggtitle("RRS Females")
```

```
#ggplot(plot_data)+geom_pointrange(aes(x = year, y = MeanRRS, ymin = lwr, ymax = upr, color = numerator), width = 0.3, position = position_dodge())+ geom_hline(aes(yintercept = 1), linetype = 2, col
```

Hard to see given the very high range in 2015. We’ll make a second plot.

```
ggplot(plot_data)+geom_errorbar(aes(x = year, y = MeanRRS, ymin = lwr, ymax = upr, color = numerator), width = 0.3, position = position_dodge())+ geom_hline(aes(yintercept = 1), linetype = 2, color = "darkgrey")+theme_bw()+geom_point(aes(x = year, y = MeanRRS, color = numerator), size = 3,  position = position_dodge(width = 0.3))+xlab("")+scale_color_manual(name = "Contrast", values = c("#66CCEE", "#CCBB44", "#AA3377"), labels = c(expression(F[1]*" / NOR"),expression("HOR / " *F[1]), "HOR / NOR" ))+ylab(expression(" "[Delta]*"RRS"))+ theme(legend.text.align = 0) +ggtitle("RRS Females")+ coord_cartesian(ylim=c(0,1.5))
```

### Results Summary

When split up by sex, HOR/NOR RRS < 1 for males in 2 of 4 years and for females in 3 of 4 years.

HOR/F1 RRS < 1 for males in 2 of 4 years and for females in 3 of 4 years.

F1/NOR RRS < 1 for males in 1 year and zero years for females.

# Differences among Offspring

We found that HORs, F1s and NOR immigrants produce different numbers of adult offspring, are there other differences in their offspring, such as age at maturity, or size ?

## AAM

The plot below can be a bit confusing so let’s be clear. These plots show the age of OFFSPRING of each of the three groups, not the age of the groups.

```
pedigree_long <- pedigree %>%
  pivot_longer(-offspring_sample_id, names_to = "parent_sex", values_to = "parent")

off_age_F12 <- F12_mmdata %>%
  left_join(select(pedigree_long, -parent_sex), by = c("sample_id" = "parent")) %>%
  left_join(select(aam_data, offspring_sample_id, age)) %>%
  rename(offspring_age = age) %>%
  left_join(select(dedup, sample_id, offspring_sex = sex, year_off = year), by = c("offspring_sample_id" = "sample_id")) %>%
  filter(!is.na(offspring_sample_id)) %>%
  mutate(offspring_sex = as.factor(offspring_sex))

ggplot(off_age_F12)+geom_bar(aes(x = generation, fill = offspring_age))+scale_fill_viridis_d()+theme_bw()+xlab("parent")
```

```
#also make proportional
ggplot(off_age_F12)+geom_bar(aes(x = generation, fill = offspring_age), position = "fill")+scale_fill_viridis_d()+theme_bw()+xlab("parent")+ylab("proportion")
```

Yes, offspring of HOR parents tend to be younger than those F1s or NOR immigrants, but is this difference stronger than what we would expect by chance alone? Sample size in NOR immigrants is looking a little small.

Age at maturity is a count variable, but it could arguably be modeled as a continuous variable as well. Let’s be sure to do it the “right” way here first. Given the way we defined age we need to recognize recognize that fish can either be 3, 4, 5 or 6 years old, and not 3.5 years old.

### AAM GLM

First we should note that there are very few age-3 or age-6 offspring. If we remove these individuals, we can fit a binomial model. In other words we will parameterize a model that predicts the probability for the different groups to produce offspring that return at age 5 vs age 4.

#### EDA and Modeling

Just like with the TLF we will be thoughtful about how to combine data across years. First let see if there is a relationship across years that we need to be concerned with.

The first question is what “year” to use. The year of parents spawned or the year the offspring returned. The latter doesn’t make a lot of sense given that our response measure is age: Because we don’t sample parents and offspring in infinite years, there will always be a bias towards young offspring in early years and old offspring in later years. For example, 2020 returning offspring need to be included, but they can only be 5 years produced in 2015 or 6 year olds in 2014. See below.

```
ggplot(off_age_F12)+geom_bar(aes(x = year_off, fill = offspring_age))+scale_fill_viridis_d()+theme_bw()+xlab("offspring year")+ylab("proportion")
```

So we should not use offspring year to control for interannual variation. Let’s look at parent year.

```
ggplot(off_age_F12)+geom_bar(aes(x = year, fill = offspring_age))+scale_fill_viridis_d()+theme_bw()+xlab("offspring year")+ylab("proportion")
```

Yes, 2015 is mostly age 4, but 2015 is also mostly HOR parents. We should be careful to look for multicollinearity and interactions.

Let’s also look at the effect of offspring sex. It would be surprising if there was not a pattern here.

```
ggplot(off_age_F12)+geom_bar(aes(x =offspring_sex, fill = offspring_age), position = "fill")+scale_fill_viridis_d()+theme_bw()+xlab("Offspring Sex")+ylab("proportion")
```

Sure enough, males return younger.

Let’s look at the relationship between year and generation and assess multicollinearity. We will assess with the GVIF.

```
off_age_F12 %<>%
  filter(offspring_age %in% c("4", "5")) 

select(off_age_F12, generation, year, offspring_age, offspring_sex) %>%
  pairs(., lower.panel = panel.cor, diag.panel = panel.hist, upper.panel = panel.smooth2)
```

```
gen_age_ind <- glm(offspring_age ~ generation + year + offspring_sex , family = "binomial", data = off_age_F12)
# is there collinearity issue (e.g. does the impact of year and generation interact)
vif(gen_age_ind)
```

```
##                   GVIF Df GVIF^(1/(2*Df))
## generation    1.259355  2        1.059344
## year          1.297145  3        1.044315
## offspring_sex 1.038094  1        1.018869
```

We know there’s some balance issues among year already (most NOR immigrants come from 2012) and this shows up in the biplot. But it doesn’t produce multicollinearity issues (GVIF is very small)

The next step is to see if there is an interaction

```
gen_age_ind <- glm(offspring_age ~ generation * year + offspring_sex*generation , family = "binomial", data = off_age_F12)
drop1(gen_age_ind, test = "Chisq")
```

LRT suggests not ( p > 0.05, delta AIC < 1), so I think we are good to include year at the start of model selection as a fixed effect and ignore year specific generation patterns.

```
gen_age_ind <- glm(offspring_age ~  year + offspring_sex*generation, family = "binomial", data = off_age_F12)
drop1(gen_age_ind, test = "Chisq")
```

```
# drop year, doesn't show up as significant, pvalue = 0.1 delta AIC <1

gen_age_ind <- glm(offspring_age ~ generation*offspring_sex , family = "binomial", data = off_age_F12)
drop1(gen_age_ind, test = "Chisq")
```

```
# interaction between generation and sex is marginal, delta AIC ~2 and p value is 0.05. This is pretty marginal. Let's take a closer look

summary(gen_age_ind)
```

```
## 
## Call:
## glm(formula = offspring_age ~ generation * offspring_sex, family = "binomial", 
##     data = off_age_F12)
## 
## Deviance Residuals: 
##     Min       1Q   Median       3Q      Max  
## -1.5936  -1.0294  -0.7775   1.2187   1.7125  
## 
## Coefficients:
##                                       Estimate Std. Error z value Pr(>|z|)    
## (Intercept)                           -0.09663    0.16628  -0.581 0.561178    
## generationF1                           1.03663    0.28858   3.592 0.000328 ***
## generationNORimmigrant                 1.01292    0.37989   2.666 0.007668 ** 
## offspring_sexM                        -0.94483    0.22405  -4.217 2.48e-05 ***
## generationF1:offspring_sexM           -0.35381    0.37300  -0.949 0.342846    
## generationNORimmigrant:offspring_sexM -1.17544    0.50352  -2.334 0.019573 *  
## ---
## Signif. codes:  0 '***' 0.001 '**' 0.01 '*' 0.05 '.' 0.1 ' ' 1
## 
## (Dispersion parameter for binomial family taken to be 1)
## 
##     Null deviance: 943.68  on 694  degrees of freedom
## Residual deviance: 858.85  on 689  degrees of freedom
## AIC: 870.85
## 
## Number of Fisher Scoring iterations: 4
```

We should prbably take this interaction into account. Final model is generation\*offspring sex. Let’s validate, summarise the fit and do some post hoc tests.

```
simulateResiduals(gen_age_ind , plot = TRUE)
```

```
## Object of Class DHARMa with simulated residuals based on 250 simulations with refit = FALSE . See ?DHARMa::simulateResiduals for help. 
##  
## Scaled residual values: 0.04899169 0.6234619 0.01499991 0.9433925 0.7450565 0.9380031 0.226312 0.4177959 0.1908391 0.4352147 0.8588953 0.172134 0.7211429 0.5541354 0.2125852 0.8872717 0.2354525 0.8101993 0.969606 0.7704067 ...
```

```
em <- emmeans(gen_age_ind, ~ generation | offspring_sex, type = "response")
contrast(em, "pairwise", type = "response")
```

```
## offspring_sex = F:
##  contrast           odds.ratio    SE  df null z.ratio p.value
##  HOR / F1                0.355 0.102 Inf    1  -3.592  0.0010
##  HOR / NORimmigrant      0.363 0.138 Inf    1  -2.666  0.0209
##  F1 / NORimmigrant       1.024 0.425 Inf    1   0.057  0.9982
## 
## offspring_sex = M:
##  contrast           odds.ratio    SE  df null z.ratio p.value
##  HOR / F1                0.505 0.119 Inf    1  -2.889  0.0108
##  HOR / NORimmigrant      1.176 0.389 Inf    1   0.492  0.8752
##  F1 / NORimmigrant       2.329 0.807 Inf    1   2.441  0.0389
## 
## P value adjustment: tukey method for comparing a family of 3 estimates 
## Tests are performed on the log odds ratio scale
```

```
em <- as.data.frame(em)
em %<>%
  mutate(offspring_sex = case_when(offspring_sex == "F" ~ "Female",
                                   TRUE ~ "Male"))

ggplot(data = em) + 
  geom_point(aes(x = generation, y=prob), size = 3)+ 
  geom_errorbar(aes(x = generation, ymin = asymp.LCL, ymax = asymp.UCL), width = 0.2)+ 
  theme_bw()+ylab("Proportion Age-5 Offspring\n vs. Age-4 Offspring")+xlab("Origin of Parent")+guides(color=guide_legend(title="Sex of Offspring")) + scale_x_discrete(labels = c( "HOR", expression(F[1]), "NOR"))+ 
  facet_grid(cols=vars(offspring_sex))
```

```
#eff1 <- predictorEffect("generation", gen_age_ind)
#effdf <- as.data.frame(eff1)
#effdf$generation <- factor(effdf$generation, levels=c("HOR", "F1", "NORimmigrant")) # relevel the genertions for a nicer plot


#ggplot(data = effdf, aes(x = (generation), y = fit))+ 
#  geom_point(position=position_dodge(width=0.3), size = 3) + 
#  geom_errorbar(aes(ymin = lower, ymax = upper), position=position_dodge(width=0.3), width = 0.1)+ylab("Proportion Age-5 Offspring\n vs. Age-4 Offspring")+xlab("Generation")+theme_bw()
```

For females we have a similar pattern to TLF, F1s and NORs produce more age 5 offspring than HORs, similar age offspring to each other. For males, F1s producemore age 5 offspring than HORs and NORs, but no other contrast is significant.

## Size

What about size? Specifically do HORs F1s and NOR immigrants produce offspring that differ in size.

First let’s take a look at the data. Can we fit a simple linear model here or do we need to incorporate covariates like we have elsewhere? Let’s look at parent year by offspring size effects.

```
#let's put together the dataset
pedigree_long <- pedigree %>%
  pivot_longer(-offspring_sample_id, names_to = "parent_sex", values_to = "parent")

off_age_F12_2 <- F12_mmdata %>%
  left_join(select(pedigree_long, -parent_sex), by = c("sample_id" = "parent")) %>%
  left_join(select(aam_data, offspring_sample_id, age)) %>%
  rename(offspring_age = age) %>%
  filter(!is.na(offspring_sample_id))

off_age_F12_2 %<>%
  left_join(select(dedup, sample_id, length_off = length, year_off = year, offspring_sex = sex), by = c("offspring_sample_id" = "sample_id")) %>%
  mutate(year_off = as.factor(as.character(year_off)),
         year_f = as.factor(year),
         offspring_sex = as.factor(offspring_sex)) %>%
  drop_na(length_off)

# sample size will be smaller because not all inds have length measurement, let's check
off_age_F12_2 %>% 
  count(generation, year) %>% 
  pivot_wider(id_cols = year, names_from = generation, values_from = n)
```

```
ggplot(off_age_F12_2)+geom_histogram(aes(x = length_off, fill = year), bins = 20, position = position_dodge())+xlab("length of offspring (cm)")+ guides(fill=guide_legend(title="Parent Year"))+theme_bw()
```

Yes, there are some very clear differences between parent years and offspring years in size. One issue compared to the age glm is that fewer individuals have length measurements, so we have less power. The sample size in 2014 and 2015 is very small. It might also be worth looking for effects within individual years if we can’t effectively control for year.

Just like the AAM GLM we should look for multicollinearity and possible year\*generation patterns before we begin.

```
size_lm <- lm(length_off ~  year_f*generation + offspring_sex*generation, data = off_age_F12_2)
Anova(size_lm , type = "II")
```

No year\*generation interaction. What about collinearity.

```
size_lm <- lm(length_off ~  year_f+generation + offspring_sex, data = off_age_F12_2)
vif(size_lm)
```

```
##                   GVIF Df GVIF^(1/(2*Df))
## year_f        1.224220  3        1.034292
## generation    1.144664  2        1.034355
## offspring_sex 1.073431  1        1.036065
```

Looking good here too.

We’ll fit a linear model with three fixed effects, parent generation, parent year, and offspring sex, and the offspring\_sex\*generation interaction check model assumptions and evaluate significance with an F-test and type II SS.

```
#no this uses the wrong Sum of Squares
#summary(lm(length_off ~ generation + year_off, data = off_age_F12_2))

#Since we'll be comparing this to the AAM results, let's also run a model to make sure we have similar results when only look at age 4 and 5 offspring.

size_lm <- lm(length_off ~  year_f*generation + offspring_sex*generation, data = filter(off_age_F12_2, offspring_age != 3, offspring_age !=6))

#plot(size_lm), no obvious issues here, let's move ahead

Anova(size_lm , type = "II")
```

```
#no this uses the wrong Sum of Squares
#summary(lm(length_off ~ generation + year_off, data = off_age_F12_2))

size_lm <- lm(length_off ~ year_f + offspring_sex*generation, data = filter(off_age_F12_2))

#plot(size_lm), no obvious issues here, let's move ahead

Anova(size_lm , type = "II")
```

```
em <- emmeans(lm(length_off ~ year_f + offspring_sex*generation , data = off_age_F12_2), ~  generation | offspring_sex, type = "response")
#contrast(em, "pairwise", type = "response")
plot(em, comparisons = TRUE)
```

```
contrast(em, "pairwise")
```

```
## offspring_sex = F:
##  contrast           estimate    SE  df t.ratio p.value
##  HOR - F1             -0.467 1.231 460  -0.379  0.9238
##  HOR - NORimmigrant   -2.591 1.402 460  -1.848  0.1553
##  F1 - NORimmigrant    -2.124 1.475 460  -1.440  0.3211
## 
## offspring_sex = M:
##  contrast           estimate    SE  df t.ratio p.value
##  HOR - F1             -1.080 0.892 460  -1.212  0.4469
##  HOR - NORimmigrant    1.742 1.084 460   1.607  0.2437
##  F1 - NORimmigrant     2.822 1.148 460   2.458  0.0381
## 
## Results are averaged over the levels of: year_f 
## P value adjustment: tukey method for comparing a family of 3 estimates
```

```
#ggplot(off_age_F12_2)+geom_histogram(aes(x = length_off, fill = generation), bins = 20, position = position_dodge())+xlab("length of offspring (cm)")+ guides(fill=guide_legend(title="Generation of Parent"))+scale_fill_manual(values = c("#4477AA", "#CCBB44", "#AA3377"))+theme_bw()
```

Results are similar in trend to age, but the confidence intervals are much wider, leading to only one significant pairwise comparisons, male F1s are bigger than male NORs.

Let’s go a little further here. Is this just a power issue? If we take the qualitative results at face value, offspring of HORs have lower age at maturity, but same size at maturity as F1s, this suggests faster growth rate and therefore size at age for first than second generation hatchery descendants. Is this true?

```
ggplot(off_age_F12_2)+geom_density(aes(x = length_off, color = generation, fill = generation), alpha = 0.5, position = position_dodge())+ facet_grid(rows = vars(offspring_age), cols = vars(offspring_sex))+scale_fill_manual(values = c("#228833", "#CCBB44", "#AA3377"))+scale_color_manual(values = c("#228833", "#CCBB44", "#AA3377"))+ggtitle("Size at Age Among Offspring")+xlab("Size at Age (cm)")
```

When we combine data across years, there’s no evidence that HOR achieve higher size at age.

```
#within years plots
ggplot(filter(off_age_F12_2, year == 2012))+geom_histogram(aes(x = length_off, color = generation, fill = generation), alpha = 0.5, position = position_dodge())+ facet_grid(rows = vars(offspring_age))+scale_fill_manual(values = c("#228833", "#CCBB44", "#AA3377"))+scale_color_manual(values = c("#228833", "#CCBB44", "#AA3377"))+ggtitle("Size at Age Among Offspring Produced in 2012")

ggplot(filter(off_age_F12_2, year == 2013))+geom_histogram(aes(x = length_off, color = generation, fill = generation), alpha = 0.5, position = position_dodge())+ facet_grid(rows = vars(offspring_age))+scale_fill_manual(values = c("#228833", "#CCBB44", "#AA3377"))+scale_color_manual(values = c("#228833", "#CCBB44", "#AA3377"))+ggtitle("Size at Age Among Offspring Produced in 2013")

ggplot(filter(off_age_F12_2, year == 2014))+geom_histogram(aes(x = length_off, color = generation, fill = generation), alpha = 0.5, position = position_dodge())+ facet_grid(rows = vars(offspring_age))+scale_fill_manual(values = c("#228833", "#CCBB44", "#AA3377"))+scale_color_manual(values = c("#228833", "#CCBB44", "#AA3377"))+ggtitle("Size at Age Among Offspring Produced in 2014")

ggplot(filter(off_age_F12_2, year == 2015))+geom_histogram(aes(x = length_off, color = generation, fill = generation), alpha = 0.5, position = position_dodge())+ facet_grid(rows = vars(offspring_age))+scale_fill_manual(values = c("#228833", "#CCBB44", "#AA3377"))+scale_color_manual(values = c("#228833", "#CCBB44", "#AA3377"))+ggtitle("Size at Age Among Offspring Produced in 2015")
```

```
#Seems pretty clear from the plots that there is no size at age difference. Let's also some anovas.
summary(aov(length_off ~ generation, data = filter(off_age_F12_2, offspring_age == 4, year == 2012)))
summary(aov(length_off ~ generation, data = filter(off_age_F12_2, offspring_age == 5, year == 2012)))
summary(aov(length_off ~ generation, data = filter(off_age_F12_2, offspring_age == 4, year == 2013)))
summary(aov(length_off ~ generation, data = filter(off_age_F12_2, offspring_age == 5, year == 2013)))
```

So, no, there are no size at age differences between offspring of the different generations. Most likely explanation of differnt results between age and size results is statistical power. Sample size is smaller for length, and varaince structure is quite different (binomial vs continuous).

# Miscellania

The sections above represent the final, concise analysis, but there are many little questions, cul de sacs, and supplemental analyses that are sometimes worth retaining for later. This section collects those. Not intended to be shared widely.

## Release Day Model

The effect of release day marginally improved the fit to the data, we should examine it alongside the final model.

```
mm_f12_wjday <- glmmTMB(tlf ~ generation + length +  jday_c+ year + (1| group ) , data = F12_mmdata, family = nbinom2) 
drop1(mm_f12_wjday, test = "Chisq")
```

```
eff1 <- predictorEffect("generation", mm_f12_wjday)
effdf <- as.data.frame(eff1)
effdf$generation <- factor(effdf$generation, levels=c("HOR", "F1", "NORimmigrant")) # relevel the genertions for a nicer plot

#note that this throws an error. w/r/t this error the glmmTMB author (Ben Bolker) states that "the predicted variances are used when computing residuals (which are Pearson residuals by default) for partial residuals plots. I think that if you're not plotting partial residuals, it doesn't matter." 
# Since we do not plot partial residuals but instead th95 CI for the predition, we are good here

ggplot(data = effdf, aes(x = (generation), y = fit))+ 
  geom_point(position=position_dodge(width=0.3), size = 3) + 
  geom_errorbar(aes(ymin = lower, ymax = upper), position=position_dodge(width=0.3), width = 0.1)+ylab("TLF")+xlab("Generation")+theme_bw()
```

```
em <- emmeans(mm_f12_wjday, "generation", weights = "cell")
plot(em, alpha = 0.05, comparisons = TRUE, adjust = "none")
```

```
contrast(em, "pairwise", adjust = "Tukey", type = "response")
```

```
##  contrast           ratio    SE   df null t.ratio p.value
##  HOR / F1           0.651 0.098 2717    1  -2.851  0.0122
##  HOR / NORimmigrant 0.580 0.091 2717    1  -3.472  0.0015
##  F1 / NORimmigrant  0.890 0.155 2717    1  -0.668  0.7818
## 
## Results are averaged over the levels of: year 
## P value adjustment: tukey method for comparing a family of 3 estimates 
## Tests are performed on the log scale
```

The results of the paper would be qualitatively the same had we chose this as our final model. RRS is less than one for both HOR/F1 and HOR/F1, but the RRS of F1/NOR = 1. Quantitatively there are some subtle differences that suggest that accounting for release day reduces the estimated fitness of F1s, but not enough to be significant or change any conclusions.

One thing we know from the big evaluation study is that the the effect of release day on fitness varies from year to year, but not enough to justify a random slopes model for year \* release day. I don’t think there is sufficient amount of data to build such a complex model, and it is unlikely to get better in future years given that downstream recycling post 2015 drastically reduces the number of NOR immigrants released above the dam.

## Carcass NOR Size

One concern with the NOR immigrants is that they are not representative of the broader McKenzie River wild population. Maybe they are late-season dispersers or strays with lower fitness? We can’t directly address this because NORs are volitional migrators, not an experimentally trap-and-hauled “wild” salmon from throughout the basin. One way to compare using available data is size. We have size estimates from wild below dam spawners from spawning ground surveys conducted thoughout the study. Since we know size and fitness are closely related, this provides at least some information that might let us know if NORs are not representative of the broader wild McKenzie population.

```
sgs_meta <- readxl::read_xlsx("../input_data/carcass_meta_data/McKenzie DNA missing_data_BoxUnableToSave_FinalDraft.xlsx", sheet = 1)

#now lets do some cleanup
sgs_meta %<>%
  mutate(length = case_when(`FL (cm)` > 111 ~ `FL (cm)`/10,
                            TRUE ~`FL (cm)` )) %>% #some lengths are in mm and some in cm, the largest cm is 111, we'll use this as a cutoff and convert 
  mutate(generation = "SGS") %>% # add a label
  mutate(year = lubridate::year(Date)) %>% #get years
  filter(year != 2011) %>% # filter years to match input data
  filter(River != "Horse Creek", River != "Lost Creek") %>% #get rid of non South Fork Tribs, just focus on below the dam and mainstem
  mutate(year = as.factor(year)) %>%
  mutate(sex = case_when(Sex == "M" ~ "M",
                         Sex == "Male" ~ "M",
                         Sex == "Female" ~ "F",
                         Sex == "F" ~ "F",
                         TRUE ~ NA_character_)) %>%
  drop_na(length) %>%
  drop_na(sex)

# now combine a with the dataset from the modeling

size_data <- F12_mmdata %>%
  select(generation, length, year, sex) %>%
  bind_rows(select(sgs_meta, generation, length, year, sex ))# %>%
#  filter(year == 2012)

size_data %>% count(generation, year) %>% pivot_wider(id_cols = year, names_from = generation, values_from = n)
```

There are 233 SF McKenzie NOR carcass samples from 2012 that have a length measurement. Since this is such a simple comparison, There may be internannual variation in length, but the sample size \* generation is pretty strongly unbalanced across years. Most carcass samples come from a year when few immigrants are sampled, 2015. Let’s lump them all together and keep in mind that we can control for year only if we assume there is no year\*generation interaction. We should maintain the possibility that NOR immigrants are different than wild spawners below the dam, regardless of the outcome of this analysis.

```
ggplot(size_data)+geom_boxplot(aes(generation, length, color = year))+theme_bw()
```

```
ggplot(size_data)+geom_boxplot(aes(generation, length, color = sex))+theme_bw()
```

```
size_aov <- aov(length~ year+ generation *  sex  , data = size_data)
Anova(size_aov, type = "II")
```

```
#TukeyHSD(size_aov)  

#predictorEffect("generation", size_aov)

em <- emmeans(size_aov, ~generation | sex)
contrast(em, "pairwise", adjust = "Tukey", type = "response")
```

```
## sex = F:
##  contrast           estimate    SE   df t.ratio p.value
##  F1 - HOR              4.769 0.554 3326   8.602  <.0001
##  F1 - NORimmigrant     1.703 0.912 3326   1.868  0.2420
##  F1 - SGS              0.581 0.648 3326   0.897  0.8066
##  HOR - NORimmigrant   -3.067 0.796 3326  -3.852  0.0007
##  HOR - SGS            -4.188 0.435 3326  -9.636  <.0001
##  NORimmigrant - SGS   -1.122 0.861 3326  -1.303  0.5610
## 
## sex = M:
##  contrast           estimate    SE   df t.ratio p.value
##  F1 - HOR              2.064 0.497 3326   4.152  0.0002
##  F1 - NORimmigrant    -2.403 0.697 3326  -3.447  0.0032
##  F1 - SGS             -7.258 0.596 3326 -12.187  <.0001
##  HOR - NORimmigrant   -4.467 0.625 3326  -7.143  <.0001
##  HOR - SGS            -9.321 0.480 3326 -19.414  <.0001
##  NORimmigrant - SGS   -4.854 0.704 3326  -6.897  <.0001
## 
## Results are averaged over the levels of: year 
## P value adjustment: tukey method for comparing a family of 4 estimates
```

```
plot(em, comparisons = TRUE)
```

In the plot above the blue bars represent confidence intervals of marginal means, and red arrows are for the pairwise Tukey comparisons among them.

For females, the only pairwise comparisons that are significant are those that include HORs (HORs always smaller).

For males, every pairwise comparison is different. HORS < F1 < NOR < SGS.

Let’s also make publication worthy plot.

```
plot_data <- as.data.frame(em)
plot_data %<>%
  mutate(generation = fct_relevel(generation, "HOR", "F1", "NORimmigrant", "SGS"),
         sex = case_when(sex == "F" ~ "Female",
                         TRUE ~ "Male")) 
  

ggplot(data = plot_data) + 
  geom_point(aes(x = generation, y=emmean), size = 3)+ 
  geom_errorbar(aes(x = generation, ymin = lower.CL, ymax = upper.CL), width = 0.2)+ 
  theme_bw()+ylab("Length (cm)")+xlab("Origin")+ scale_x_discrete(labels = c( "HOR", expression(F[1]), "NOR", "Carcass"))+ 
  facet_grid(cols=vars(sex))
```

## AAM TLF

```
age_mm_data <- F12_mmdata %>% left_join(select(aam_data, offspring_sample_id, age), by = c("sample_id" = "offspring_sample_id")) %>% drop_na(age)

mm_age <- glmmTMB(tlf ~ jday_c + sex + age + year+ age*sex + length + (1|group) , data = age_mm_data, family = nbinom2)
drop1(mm_age, test = "Chisq")
# drop the interaction
mm_age <- glmmTMB(tlf ~ jday_c + sex + age + year + length+ (1|group) , data = age_mm_data, family = nbinom2)
drop1(mm_age, test = "Chisq")
#drop sex
mm_age <- glmmTMB(tlf ~ jday_c + age + year + (1|group) , data = age_mm_data, family = nbinom2)
drop1(mm_age, test = "Chisq")
#drop jday
mm_age <- glmmTMB(tlf ~  age + year + (1|group) , data = age_mm_data, family = nbinom2)
drop1(mm_age, test = "Chisq")

#validate fit
simulateResiduals(mm_age, plot = TRUE)

#estimate marginal means
em <- emmeans(mm_age, "age_f")
contrast(em, "pairwise", type = "response")
```

## Release Location

We know a priori that there are no fitness differences associated with release location in this reintroduction, but the readers/reviewers do not. Unmarked fish are released only at one location, whereas HORs are released at several. Let’s be very conservative and only compare fish released at the single shared site. Then we’ll refit the final model and see how sensitive the parameter estimates are.

```
ggplot(F12_mmdata)+geom_bar(aes(x = release, fill = generation), alpha = 0.8) + scale_fill_manual(values = c("#228833", "#CCBB44", "#AA3377"))+ theme_bw()+xlab("Release Location")
```

```
mm_f12_hr <- glmmTMB(tlf ~ generation + length +   year + sex+ (1| group ) , data = filter(F12_mmdata, release == "Hard Rock"), family = nbinom2) 
drop1(mm_f12_hr, test = "Chisq")
```

```
summary(mm_f12_hr)
```

```
##  Family: nbinom2  ( log )
## Formula:          tlf ~ generation + length + year + sex + (1 | group)
## Data: filter(F12_mmdata, release == "Hard Rock")
## 
##      AIC      BIC   logLik deviance df.resid 
##   1878.0   1930.1   -929.0   1858.0     1344 
## 
## Random effects:
## 
## Conditional model:
##  Groups Name        Variance Std.Dev.
##  group  (Intercept) 0.01976  0.1406  
## Number of obs: 1354, groups:  group, 117
## 
## Dispersion parameter for nbinom2 family (): 0.765 
## 
## Conditional model:
##                         Estimate Std. Error z value Pr(>|z|)    
## (Intercept)            -6.955009   0.729521  -9.534  < 2e-16 ***
## generationF1            0.627948   0.184151   3.410 0.000650 ***
## generationNORimmigrant  0.811861   0.215782   3.762 0.000168 ***
## length                  0.064994   0.008966   7.249 4.19e-13 ***
## year2013                0.982151   0.172824   5.683 1.32e-08 ***
## year2014               -0.056827   0.227373  -0.250 0.802641    
## year2015                0.024148   0.257947   0.094 0.925413    
## sexM                   -0.042714   0.126121  -0.339 0.734853    
## ---
## Signif. codes:  0 '***' 0.001 '**' 0.01 '*' 0.05 '.' 0.1 ' ' 1
```

```
em <- emmeans(mm_f12_hr, "generation", weights = "cell")
rrs_model <- contrast(em, "pairwise", adjust = "Tukey", type = "response")
rrs_model <- as.data.frame(rrs_model) 
 
ggplot(data = rrs_model, aes(x = contrast, y = ratio))+ 
  geom_point(position=position_dodge(width=0.3), size = 3) + 
  geom_errorbar(aes(ymin = ratio - SE, ymax = ratio + SE), position=position_dodge(width=0.3), width = 0.1)+ylab(expression(" "[model]*"RRS"))+xlab("Contrast") + 
  theme_bw()+theme(axis.text.x = element_text(size = 16, color = "black"), axis.title = element_text(size = 16), axis.text.y = element_text(size = 12, color = "black")) + 
  scale_x_discrete(labels = c(expression(F[1]*" / NOR"),expression("HOR / " *F[1]), "HOR / NOR")) + 
  geom_hline(aes(yintercept = 1), linetype = 2, color = "darkgrey")+ ylim(0, 1.2)
```

Nearly identical results, even when we exclude about 3/4 of HORs released at other sites.

## Broodstock Selection

One concern we’ve had since the beginning of this project is that hatchery operators are likely selecting the “best” fish for broodstock and leaving the remainders for outplanting. Similarly, hatchery outplants have to be transported longer distance than individual collected at the trap. These diffences may be responsible for the fitness difference we observe between HORs and wild-born salmon.

Fortunately, we can address this concern. A relatively small number of HOR salmon are collected at the Cougar Trap (n = 71 in the 2012 - 2015 dataset, and n = 218 in the 2010 = 2015 dataset). Let’s ask the question, are there any differences between HOR outplants (from the hatchery) and HORs from the trap?

First size:

```
#all years
lm2 <- (lm(length ~ type, data = filter(dedup, origin == "HOR")))

#just the individuals from this paper
lm2 <- (lm(length ~  year + sex + jday_c + type, data = filter(F12_mmdata, origin == "HOR")))

Anova(lm2 , type = "II")
```

```
# validate
#plot(lm(length ~ length +  year + sex + jday_c + type, data = filter(F12_mmdata, origin == "HOR")))
# looks good other than a very small number of jacks, potentially causing issues, not enough to fail any validation, but let's remove these and test again to be sure

lm2 <- (lm(length ~  year + sex + jday_c + type, data = filter(F12_mmdata, origin == "HOR", length > 40)))

Anova(lm2 , type = "II")
```

```
#plot(lm2), still looks good

Anova(size_lm , type = "II")
```

No difference in size.

What about fitness?

```
# 
filter(F12_mmdata, origin == "HOR") %>% 
  group_by(type) %>%
  summarise(mean_tlf = mean(tlf))
```

Cougar Trap HORs actually have slightly LOWER fitness.

Let’s get more sophisticated and build a fitness model that incorporates covariates. We let’s build a model using all predictors that make sense (size, sex, year, release day), plus the hatchery vs cougar effect, then do model selection. Let’s not do the random effect as sample size within levels for Cougar Trap individuals are small.

```
mm_f12_HOR <- glmmTMB(tlf ~  length +  year + sex + jday_c + type , data = filter(F12_mmdata, origin == "HOR"), family = nbinom2) 
drop1(mm_f12_HOR, test = "Chisq") 

mm_f12_HOR <- glmmTMB(tlf ~  length +  year + sex + type , data = filter(F12_mmdata, origin == "HOR"), family = nbinom2) 
drop1(mm_f12_HOR, test = "Chisq") 

mm_f12_HOR <- glmmTMB(tlf ~  length +  year +  type, data = filter(F12_mmdata, origin == "HOR"), family = nbinom2) 
drop1(mm_f12_HOR, test = "Chisq") 

# validate
simulateResiduals(mm_f12_HOR, plot = TRUE)


em <- emmeans(mm_f12_HOR, "type", type = "response")
#plot(em)
```

Nope, still no difference in fitness. Power is probably somewhat low given the sample size/width of CL for trap HORs, but the interval is still less than the lower bounds for NORs/F1s.

## Final Figures

### Coastwide

In this section we plot key results from above for a powerpoint presentation. Eval flag is set to false for all of these so they should not produce anything.

```
# First the TLF figure

eff1 <- predictorEffect("generation", mm_f12)
effdf <- as.data.frame(eff1)
effdf$generation <- factor(effdf$generation, levels=c("HOR", "F1", "NORimmigrant")) # relevel the genertions for a nicer plot

#note that this throws an error. w/r/t this error the glmmTMB author (Ben Bolker) states that "the predicted variances are used when computing residuals (which are Pearson residuals by default) for partial residuals plots. I think that if you're not plotting partial residuals, it doesn't matter." 
# Since we do not plot partial residuals but instead th95 CI for the predition, we are good here

ggplot(data = effdf, aes(x = (generation), y = fit))+ 
  geom_point(position=position_dodge(width=0.3), size = 3) + 
  geom_errorbar(aes(ymin = lower, ymax = upper), position=position_dodge(width=0.3), width = 0.1)+ylab("TLF")+xlab("Generation")+theme_bw()+theme(axis.text = element_text(size = 16, color = "black"))

ggplot(data = effdf)+geom_bar(aes(x = generation, y = fit, color = generation, fill = generation), stat = "identity")+geom_errorbar(aes(x = generation, ymin = lower, ymax = upper), position=position_dodge(width=0.3), width = 0.1)+ylab("TLF")+xlab("Generation")+theme_bw()+theme(axis.text.x = element_text(size = 18, color = "black"), axis.text.y = element_text(size = 14, color = "black"), axis.title = element_text(size = 18))+scale_x_discrete(labels = c( "HOR", expression(F[1]), "NOR"))+scale_fill_manual(values = c("#228833", "#CCBB44", "#AA3377"))+scale_color_manual(values = c("#228833", "#CCBB44", "#AA3377"))+guides(color = FALSE, fill =FALSE)
```

Yet another way to plot this would be as an RRS.

```
em <- emmeans(mm_f12, "generation", weights = "cell")
rrs_model <- contrast(em, "pairwise", adjust = "Tukey", type = "response")
rrs_model <- as.data.frame(rrs_model) 
 
ggplot(data = rrs_model, aes(x = contrast, y = ratio))+ 
  geom_point(position=position_dodge(width=0.3), size = 3) + 
  geom_errorbar(aes(ymin = ratio - SE, ymax = ratio + SE), position=position_dodge(width=0.3), width = 0.1)+ylab(expression(" "[model]*"RRS"))+xlab("Contrast") + 
  theme_bw()+theme(axis.text.x = element_text(size = 16, color = "black"), axis.title = element_text(size = 16), axis.text.y = element_text(size = 12, color = "black")) + 
  scale_x_discrete(labels = c(expression(F[1]*" / NOR"),expression("HOR / " *F[1]), "HOR / NOR")) + 
  geom_hline(aes(yintercept = 1), linetype = 2, color = "darkgrey")+ ylim(0, 1.2)
```

```
pedigree_long <- pedigree %>%
  pivot_longer(-offspring_sample_id, names_to = "parent_sex", values_to = "parent")

off_age_F12 <- F12_mmdata %>%
  left_join(select(pedigree_long, -parent_sex), by = c("sample_id" = "parent")) %>%
  left_join(select(aam_data, offspring_sample_id, age)) %>%
  rename(offspring_age = age) %>%
  filter(!is.na(offspring_sample_id)) %>%
  left_join(select(dedup, sample_id, off_sex = sex), by = c("offspring_sample_id" = "sample_id"))

ggplot(off_age_F12)+facet_grid(cols = vars(off_sex))+geom_bar(aes(x = generation, fill = offspring_age))+scale_fill_viridis_d(name = "Offspring\nAge")+theme_bw()+xlab("Parent Generation")+scale_x_discrete(labels = c( "HOR", expression(F[1]), "NOR"))+ylab("Count")+theme(axis.text.x = element_text(size = 18, color = "black"), axis.text.y = element_text(size = 14, color = "black"), axis.title = element_text(size = 18), legend.text = element_text(size = 14), , legend.title = element_text(size = 14))

#also make proportional

ggplot(off_age_F12)+facet_grid(cols = vars(off_sex))+geom_bar(aes(x = generation, fill = offspring_age), position = "fill")+scale_fill_viridis_d(name = "Offspring\nAge")+theme_bw()+xlab("Parent Generation")+scale_x_discrete(labels = c( "HOR", expression(F[1]), "NOR"))+ylab("Proportion")+theme(axis.text.x = element_text(size = 18, color = "black"), axis.text.y = element_text(size = 14, color = "black"), axis.title = element_text(size = 18), legend.text = element_text(size = 14), , legend.title = element_text(size = 14))
```

```
em <- emmeans(gen_age_ind, ~ generation | offspring_sex, type = "response")
contrast(em, "pairwise", type = "response")

pd <- as.data.frame(em)

pd %<>%
  mutate(offspring_sex = case_when(offspring_sex == "F" ~ "Female",
                                   offspring_sex == "M" ~ "Male"))
  

ggplot(pd) + 
  geom_point(aes(x = generation, y=prob), size = 3)+ 
  geom_errorbar(aes(x = generation, ymin = asymp.LCL, ymax = asymp.UCL), width = 0.2)+ 
  theme_bw()+ylab("Proportion Age-5 Offspring\n vs. Age-4 Offspring")+xlab("Generation of Parent")+guides(color=guide_legend(title="Sex of Offspring")) + scale_x_discrete(labels = c( "HOR", expression(F[1]), "NOR"))+ theme(axis.text.x = element_text(size = 16, color = "black"), axis.text.y = element_text(size = 10, color = "black"), axis.title = element_text(size = 14), legend.text = element_text(size = 12), , legend.title = element_text(size = 12))+
  facet_grid(cols=vars(offspring_sex))+theme(strip.text.x = element_text(size = 12))
```

### Paper

```
ggplot(data = F12_mmdata)+geom_density(aes(x = jday, fill = generation, color = generation), alpha = 0.4)+theme_bw()+scale_fill_manual(values = c("#228833", "#CCBB44", "#AA3377"),labels = c( "HOR", expression(F[1]), "NOR"))+scale_color_manual(values = c("#228833", "#CCBB44", "#AA3377"), labels = c( "HOR", expression(F[1]), "NOR"))+xlab("Julian Day of Release")+theme(legend.title=element_blank(), legend.text.align = 0)+ylab("Density")
```

```
eff1 <- predictorEffect("generation", mm_f12)
```

```
## Warning in Effect.glmmTMB(ans, mod, x.var = 1, xlevels = xlevels, ...):
## overriding variance function for effects: computed variances may be incorrect
```

```
effdf <- as.data.frame(eff1)
effdf$generation <- factor(effdf$generation, levels=c("HOR", "F1", "NORimmigrant")) # relevel the genertions for a nicer plot

#note that this throws an error. w/r/t this error the glmmTMB author (Ben Bolker) states that "the predicted variances are used when computing residuals (which are Pearson residuals by default) for partial residuals plots. I think that if you're not plotting partial residuals, it doesn't matter." 
# Since we do not plot partial residuals but instead th95 CI for the predition, we are good here

ggplot(data = effdf, aes(x = (generation), y = fit))+ 
  geom_point(position=position_dodge(width=0.3), size = 3) + 
  geom_errorbar(aes(ymin = lower, ymax = upper), position=position_dodge(width=0.3), width = 0.1)+ylab("TLF")+xlab("Origin")+theme_bw()+scale_x_discrete(labels = c( "HOR", expression(F[1]), "NOR"))+theme(text = element_text(size = 16))
```
